# Alterations of the Human Gut Microbiome in Chronic Kidney Disease

**DOI:** 10.1002/advs.202001936

**Published:** 2020-09-02

**Authors:** Zhigang Ren, Yajuan Fan, Ang Li, Quanquan Shen, Jian Wu, Lingyan Ren, Haifeng Lu, Suying Ding, Hongyan Ren, Chao Liu, Wenli Liu, Dan Gao, Zhongwen Wu, Shiyuan Guo, Ge Wu, Zhangsuo Liu, Zujiang Yu, Lanjuan Li

**Affiliations:** ^1^ Department of Infectious Diseases The First Affiliated Hospital of Zhengzhou University Zhengzhou 450052 China; ^2^ Gene Hospital of Henan Province Precision Medicine Center The First Affiliated Hospital of Zhengzhou University Zhengzhou 450052 China; ^3^ Department of Nephrology The First Affiliated Hospital of Zhengzhou University Zhengzhou 450052 China; ^4^ Health Management Center The First Affiliated Hospital of Zhengzhou University Zhengzhou 450052 China; ^5^ Department of Nephrology, Zhejiang Provincial People's Hospital People's Hospital of Hangzhou Medical College Hangzhou Zhejiang 310014 China; ^6^ Department of Nephrology Chun'an First People's Hospital Hangzhou Zhejiang 311770 China; ^7^ College of Public Health Zhengzhou University Zhengzhou 450052 China; ^8^ Department of Nephrology The First Affiliated Hospital of Huzhou Teachers College The First People's Hospital of Huzhou Huzhou Zhejiang 313000 China; ^9^ State Key Laboratory for Diagnosis and Treatment of Infectious Disease National Clinical Research Center for Infectious Diseases Collaborative Innovation Center for Diagnosis and Treatment of Infectious Diseases The First Affiliated Hospital School of Medicine Zhejiang University Hangzhou 310003 China; ^10^ Shanghai Mobio Biomedical Technology Co., Ltd. Shanghai 201111 China; ^11^ Clinical Laboratory Diagnostics, Medical Technology College Beihua University Jilin 132013 China

**Keywords:** chronic kidney disease, gut microbiome, microbial markers, non‐invasive diagnostic tools

## Abstract

Gut microbiota make up the largest microecosystem in the human body and are closely related to chronic metabolic diseases. Herein, 520 fecal samples are collected from different regions of China, the gut microbiome in chronic kidney disease (CKD) is characterized, and CKD classifiers based on microbial markers are constructed. Compared with healthy controls (HC, *n* = 210), gut microbial diversity is significantly decreased in CKD (*n* = 110), and the microbial community is remarkably distinguished from HC. Genera *Klebsiella* and *Enterobacteriaceae* are enriched, while *Blautia* and *Roseburia* are reduced in CKD. Fifty predicted microbial functions including tryptophan and phenylalanine metabolisms increase, while 36 functions including arginine and proline metabolisms decrease in CKD. Notably, five optimal microbial markers are identified using the random forest model. The area under the curve (AUC) reaches 0.9887 in the discovery cohort and 0.9512 in the validation cohort (49 CKD vs 63 HC). Importantly, the AUC reaches 0.8986 in the extra diagnosis cohort from Hangzhou. Moreover, *Thalassospira* and *Akkermansia* are increased with CKD progression. Thirteen operational taxonomy units are correlated with six clinical indicators of CKD. In conclusion, this study comprehensively characterizes gut microbiome in non‐dialysis CKD and demonstrates the potential of microbial markers as non‐invasive diagnostic tools for CKD in different regions of China.

## Graphical abstract

Compared with healthy controls, gut microbial diversity in CKD is significantly reduced, *Klebsiella* and *Akkermansia* are significantly increased, *Roseburia* and *Faecalibacterium* are significantly reduced, and the predictive function of gut microbiota such as ascorbate metabolism and lipopolysaccharide biosynthesis is significantly enhanced. *Akkermansia* increases along with the progression of CKD, which is positively correlated with serum creatinine and blood urea nitrogen, and negatively correlated with estimated glomerular filtration rate, and could be used as a therapeutic target to improve the prognosis of CKD. Importantly, gut microbial markers have strong diagnostic potential for CKD and achieve cross‐regional validation, which can be used as a non‐invasive diagnostic tool for CKD.

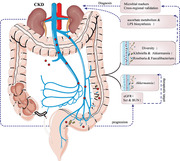

## 1 Introduction

Chronic kidney disease (CKD) is a general term for heterogeneous disorders affecting the structure and function of the kidneys^[^ ^1^ ^]^ and is associated with significant rates of morbidity, mortality, and healthcare costs.^[^ ^2^ ^]^ The mean global prevalence of CKD has been estimated at 13.4%.^[^ ^3^ ^]^ CKD progression to end‐stage renal disease (ESRD) often requires an expensive renal replacement therapy, such as hemodialysis, peritoneal dialysis, or kidney transplantation. The annual mortality rate of dialysis patients is as high as 10–20%.^[^ ^4^ ^]^ Owing to the lack of visible clinical manifestations in the early stages, most patients with CKD advance to the stage of renal failure at the time of treatment, with a poor prognosis. Therefore, to improve the prognosis of CKD patients, it is essential to search for novel diagnostic markers and therapeutic targets for CKD.

Gut microecosystem is the biggest microecosystem in the human body and plays an important role in human health and diseases.^[^ ^5^ ^]^ In recent years, increasing attention has been paid to the role of the gut microbiome in CKD. The gut‐derived uremic toxins, such as P‐cresyl sulfate (PCS), indoxyl sulfate (IS), and trimethylamine N‐oxide (TMAO), have been implicated in the progression of CKD and an increased cardiovascular risk.^[^ ^6^ ^]^ The decline of renal function has been linked to the increase in the concentration of PCS and IS.^[^ ^7^ ^]^ Thus, gut microbiome is closely related to CKD. In 2012, Viziri et al.^[^ ^8^ ^]^ found that uremia profoundly alters the composition of the gut microbiome, and there was a significant difference in the abundance of 190 bacterial operational taxonomic units (OTUs) between the ESRD and control groups. Nevertheless, the characteristics of gut microbiome in patients with non‐dialysis CKD have been rarely reported.

The diagnostic potential of gut microbiome for type 2 diabetes,^[^ ^9^ ^]^ autoimmune hepatitis,^[^ ^10^ ^]^ and early hepatocellular carcinoma^[^ ^11^ ^]^ has been confirmed by compelling studies. But, the diagnosis potential of the gut microbiome for CKD needs to be further evaluated. In this study, we prospectively collected 520 fecal samples from different parts of China, of which 503 samples were subjected to Miseq sequencing, and 489 samples were included for analysis. In the discovery cohort, we characterized the gut microbiome in the 210 healthy controls and 110 CKD from Zhengzhou and constructed a CKD classifier. Then, we verified the diagnostic efficacy of the CKD classifier in both the validation cohort and the independent diagnosis cohort. Furthermore, we characterized the gut microbiome in different clinical stages of CKD.

## 2 Results

**Figure 1:**
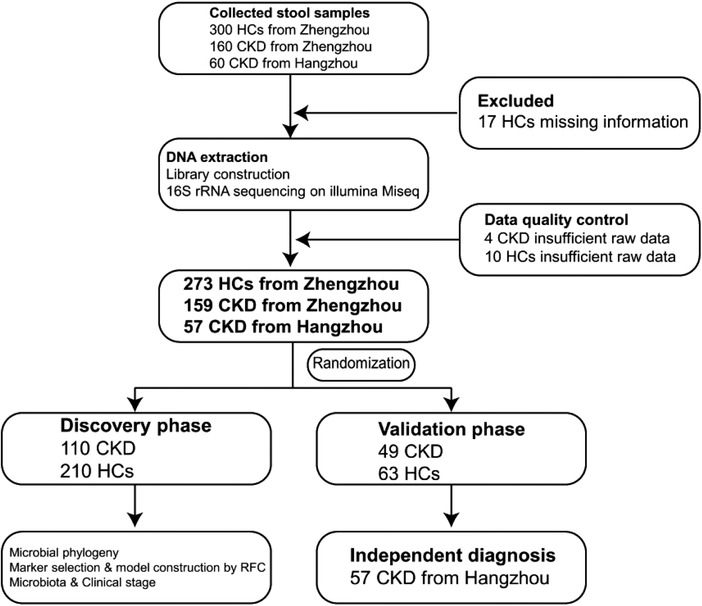
Study design and flow diagram. A total of 520 fecal samples from different parts of China were collected prospectively. After a rigorous diagnosis and exclusion procedures, 159 CKD and 273 HC samples from Zhengzhou, China, and 57 CKD samples from Hangzhou, China, were included. All samples from Zhengzhou were randomly divided into the discovery cohort and the validation cohort. In the discovery cohort, we characterized gut microbiome between 110 CKD and 210 HC and identified the microbial markers and constructed a CKD classifier by a random forest classifier model between CKD and HC. In the validation cohort, we validated the diagnosis efficacy of CKD classifier in 49 CKD and 63 HC. Finally, 57 CKD from Hangzhou served as an independent diagnostic cohort to verify the diagnostic efficacy of CKD classifier. CKD, chronic kidney disease; HC, healthy controls; RFC, random forest classifier model.

A total of 520 fecal samples from different parts of China were collected prospectively. After rigorous diagnosis and exclusion procedures, 489 fecal samples were included for analysis, including 159 CKD and 273 healthy controls from Zhengzhou, and 57 CKD from Hangzhou. The samples from Zhengzhou were randomly divided into a discovery cohort and a validation cohort (**Figure** **1**). Within the discovery cohort, we characterized the gut microbiome in 210 healthy controls and 110 patients with CKD, identified the key microbial markers, and constructed a CKD classifier using a random forest model. Within the validation cohort, the diagnosis efficacy of the CKD classifier was verified in 49 patients with CKD and 63 healthy controls. Finally, the independent diagnosis efficacy of the CKD classifier was verified in 57 patients with CKD from Hangzhou. The 110 samples of CKD patients in the discovery cohort were divided into three groups according to the clinical stages of CKD. We characterized the gut microbiome of patients in these three groups.

### 2.1 Clinical Information of the Participants

In the discovery cohort and the validation cohort, the gender, age, and body mass index (BMI) of CKD patients and healthy controls were matched (**Table** **1**). Compared with the healthy controls, serum levels of white blood cells, creatinine (SCr), blood urea nitrogen (BUN), uric acid, and the total cholesterol were significantly increased in CKD patients, and the estimated glomerular filtration rate (eGFR), serum levels of red blood cells, and albumin (ALB) levels were significantly decreased in CKD patients (Table 1). The details are listed in the online supplementary data S1.

**Table 1:** Clinical characteristics of participants in the discovery and validation cohort. Continuous variables were expressed as means ± standard deviations or median (interquartile ranges). Categorical variables were expressed as percentages. Continuous variables were compared using Student s t‐test or Wilcoxon rank‐sum test, and categorical variables were compared using Chi‐square test or Fisher’s exact test

Clinical indices		Discovery (*n* = 320)		*p*‐value	Validation (*n* = 112)		*p*‐value
		Control (*n* = 210)	CKD (*n* = 110)		Control (*n* = 63)	CKD (*n* = 49)	
Age (year)		50.02 ± 4.56	51.75 ± 14.60	0.229	46.24 ± 7.66	50.20 ± 16.09	0.116
Gender(Female/Male)		105/105	50/60	0.440	30/33	20/29	0.472
BMI		13.47 ± 2.22	13.35 ± 1.37	0.547	23.18 ± 2.13	23.13 ± 1.68	0.894
WBC [× 10^9^ per L]		5.63 ± 1.20	7.32 ± 6.21	<0.01	5.93 ± 1.24	6.71 ± 2.11	<0.05
RBC [× 10^12^ per L]		4.64 ± 0.42	3.76 ± 1.35	<0.001	4.71 ± 0.41	3.83 ± 0.80	<0.001
Hemoglobin [g L^−1^]		ND	115.08 ± 33.63	–	ND	116.61 ± 26.95	–
Platelet [× 10^9^ per L]		220.37 ± 41.77	209.02 ± 74.70	0.142	219.97 ± 37.03	219.45 ± 83.96	0.968
24h UTP(g)		ND	3.16 ± 3.56	–	ND	3.83 ± 4.55	–
ALB[g L^−1^]		48.00 ± 2.63	37.50 ± 8.10	<0.001	47.59 ± 2.77	37.51 ± 9.11	<0.001
BUN [mmol L^−1^]		4.58 ± 1.02	16.78 ± 10.24	<0.001	4.68 ± 1.04	13.24 ± 9.37	<0.001
SCr [umol L^−1^]		67.62 ± 12.93	316.98 ± 239.64	<0.001	67.95 ± 13.65	277.74 ± 294.28	<0.001
Uric acid [umol L^−1^]		284.32 ± 76.44	408.70 ± 123.94	<0.001	292.24 ± 67.95	362.90 ± 126.34	<0.001
eGFR		104.29 ± 9.78	37.18 ± 35.94	<0.001	103.38 ± 10.75	57.83 ± 63.85	<0.001
T‐chol [mmol L^−1^]		3.45 ± 1.77	5.10 ± 1.84	<0.001	3.69 ± 1.63	5.11 ± 1.60	<0.001
TG [mmol L^−1^]		1.71 ± 0.87	1.98 ± 1.71	0.141	1.61 ± 1.01	1.89 ± 1.03	0.167
Phosphate [mmol L^−1^]		ND	2.95 ± 16.27	–	ND	1.34 ± 0.43	–
Hypertension		NO	76 (69.09%)	–	NO	29 (59.18%)	–
CKD clinical stage	Stages 1–2	NO	26 (23.64%)	–	NO	16 (32.65%)	–
	Stages 3–4	NO	36 (32.73%)	–	NO	18 (36.73%)	–
	Stage 5	NO	48 (43.64%)	–	NO	15 (30.61%)	–

Abbreviations: CKD, chronic kidney disease; BMI, body mass index; WBC, white blood cells; RBC, red blood cells; 24h UTP, 24h urine protein quantitation; ALB, albumin; BUN, blood Urea nitrogen; SCr, serum creatinine; eGFR, estimated glomerular filtration rate; T‐chol, total cholesterol; TG, triglyceride; ND, no detection.

### 2.2 Gut Microbial Diversity of CKD Was Decreased

**Figure 2:**
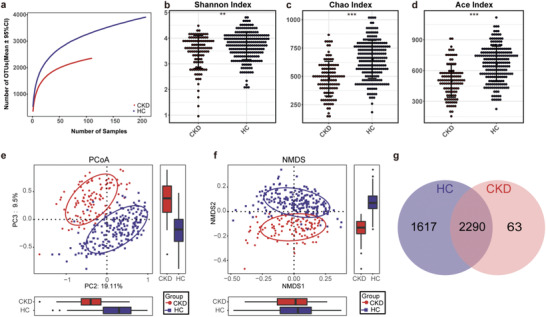
Gut microbial diversity of patients with non‐dialysis CKD was decreased. a) The rarefaction analysis between the number of samples and the number of OTUs. As the number of samples increased, the number of OTUs approached saturation in CKD (*n* = 110) and HC (*n* = 210). Compared with the HC, the number of OTUs in CKD was decreased significantly. As estimated by b) the Shannon index, c) the Chao index, and d) the Ace index, gut microbial diversity was significantly decreased in CKD (*n* = 110) compared with that in the HC (*n* = 210) (*p* < 0.01, *p* < 0.001, and *p* < 0.001, respectively). e) The PCoA and f) the NMDS based on OTUs distribution showed that the gut taxonomic composition was significantly different between CKD (*n* = 110) and HC (*n* = 210). g) A Venn diagram displaying the overlaps between groups showed that 2290 of the total number of 3970 OTUs were shared in both groups, while 63 were unique for CKD (*n* = 110). *, *p* <0.05; **, *p*<0.01; *** *p*<0.001. CKD, chronic kidney disease; HC, healthy controls; OTUs, operational taxonomic units; PCoA, principal coordinate analysis; NMDS, non‐metric multidimensional scaling.

In the discovery cohort, a rarefaction analysis showed that the number of OTUs richness nearly approached saturation in both groups as the number of samples increased, and it was significantly decreased in CKD (*n* = 110) compared with that in the healthy controls (*n* = 210) (**Figure** **2**a). The quality of the data has been shown in Figure S1a–c, Supporting Information. As estimated by the Shannon index (Figure 2b), the Chao index (Figure 2c), and the Ace index (Figure 2d), the gut microbial diversity was significantly reduced in CKD compared to the healthy controls (*p* < 0.01, *p* < 0.001, and *p* < 0.001, respectively, online supplementary data S2). And compared with healthy controls, the observed OTUs in CKD were also significantly decreased (*p* < 0.001, Figure S1d, Supporting Information).

In addition, the non‐metric multidimensional scaling (NMDS) analysis, the principal coordinate analysis (PCoA), and the principal component analysis (PCA) based on distribution of the OTUs were conducted to illustrate the microbiome space of different samples. The gut microbiome composition was significantly different between CKD and healthy controls (Figure 2e,f and Figures S2–S4, Supporting Information). As shown in a Venn diagram showing the overlaps between groups, the total abundance of OTUs was 3970, and 2290 OTUs were shared in both groups (Figure 2g). Noteworthy, 63 OTUs were unique to CKD. The key 47 OTUs between the two groups were selected, and their relative abundance and distribution were presented in a heatmap (Figure S5, Supporting Information, online supplementary data S3). As shown in the heatmap, 6 OTUs were significantly enriched in CKD, while 41 OTUs were significantly enriched in the healthy controls.

### 2.3 Phylogenetic Profiles of the Gut Microbiome in CKD

**Figure 3:**
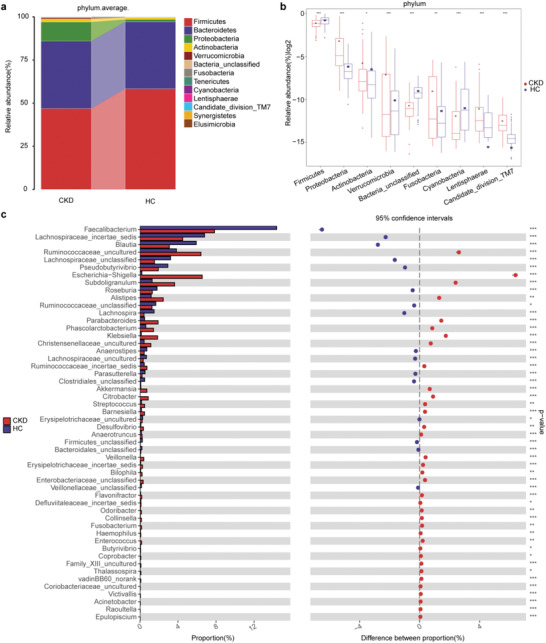
Phylogenetic profiles of the gut microbiome between CKD (*n* = 110) and HC (*n* = 210). a) Average compositions and relative abundance of the bacterial community in both groups at the phylum level. b) Compared with HC (*n* = 210), five phyla were significantly enriched, whereas four phyla were significantly reduced in CKD (*n* = 110) (all *p* < 0.05). c) Thirty‐six genera were significantly enriched, while 16 genera were significantly reduced in CKD (*n* = 110) versus HC (*n* = 210) (all *p* < 0.05). *, *p* < 0.05, **, *p* < 0.01, ***, *p* < 0.001. CKD, chronic kidney disease; HC, healthy controls.

In the discovery cohort, we further analyzed taxonomic composition and alterations of the gut microbiome in CKD. The composition and abundance of the bacterial community in each sample at the phylum and the genus levels have been shown in Figures S6 and S7, Supporting Information, respectively (online supplementary data S4 & S6). Average compositions and relative abundance of the bacterial community in both groups at the phylum and the genus levels have been shown in **Figure** **3**a and Figure S8, Supporting Information, respectively. Five phyla including *Proteobacteria, Actinobacteria*, and *Fusobacteria* were significantly enriched, while four phyla including *Firmicutes, Verrucomicrobia*, and *Bacteria unclassified* were significantly reduced in CKD versus healthy controls (all *p* < 0.05, Figure 3b, online supplementary data S5). Thirty‐six genera including *Klebsiella, Desulfovibrio*, and *Veillonella* were significantly enriched, whereas 16 genera including *Blautia, Roseburia*, and *Lachnospira* were significantly reduced in CKD compared with those in the healthy controls (all *p* < 0.05, Figure 3c, online supplementary data S7).

Furthermore, we compared the gut microbial composition between CKD and healthy controls at the class, the order, and the family levels. The abundance and composition of the bacterial community in each sample at the three levels are shown in Figures S9, S12, and S15, Supporting Information, respectively. The average composition and relative abundance of the bacterial community in both groups at the three levels are shown in Figures S10, S13, and S16, Supporting Information. At the class level, eight bacterial populations including *Actinobacteria*, *Bacilli*, and *Fusobacteria* were significantly enriched, whereas five bacterial populations including *Clostridia, Verrucomicrobia*, and *Cyanobacteria* were significantly reduced in CKD versus those in the healthy controls (all *p* < 0.05, Figure S11, Supporting Information). At the order level, nine bacterial populations including *Lactobacillales*, *Coriobacteriales*, and *Victivallales* were significantly enriched, whereas five bacterial populations including *Clostridiales*, *Burkholderiales*, and *Verrucomicrobiales* were significantly reduced in CKD versus those in the healthy controls (all *p* < 0.05, Figure S14, Supporting Information). At the family level, 16 bacterial populations including *Coriobacteriaceae*, *vadinBB60*, and *Victivallaceae* were significantly enriched, while 8 bacterial populations including *Lachnospiraceae*, *Alcaligenaceae*, and *Moraxellaceae* were significantly reduced in CKD versus those in the healthy controls (all *p* < 0.05, Figure S17, Supporting Information).

### 2.4 Crucial Bacteria and Microbial Functions Related to CKD

**Figure 4:**
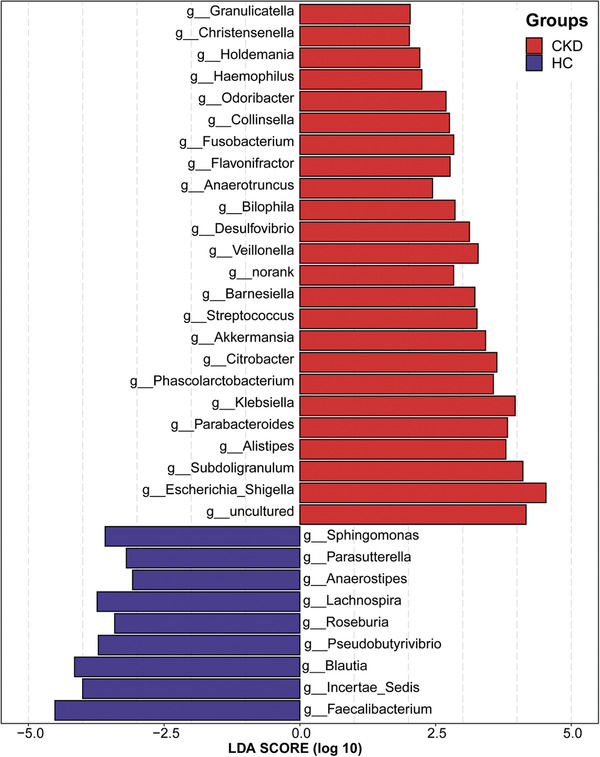
Crucial bacteria of gut microbiome related to CKD. Based on the LDA selection, 24 genera were significantly enriched, while 9 genera were significantly reduced in CKD (*n* = 110) compared with HC (*n* = 210) (all *p* < 0.01). CKD, chronic kidney disease; HC, healthy controls; LDA, linear discriminant analysis.

Linear discriminant analysis effect size (LEfSe) was used to show the maximum difference of the microbial structures in healthy controls versus those in CKD, to determine the specific bacterial taxa and predominant bacteria related to CKD. The phylogenetic profile of the specific bacterial taxa and the major bacteria associated with CKD has been shown in Figure S18, Supporting Information. Based on the linear discriminant analysis (LDA) selection, 24 genera including *Granulicatella*, *Christensenella*, and *Holdemania* were significantly enriched, while 9 genera including *Faecalibacterium*, *Incertae Sedis*, and *Blautia* were significantly reduced in CKD compared with those in the healthy controls (*p* < 0.01, **Figure** **4**, online supplementary data S8).

**Figure 5:**
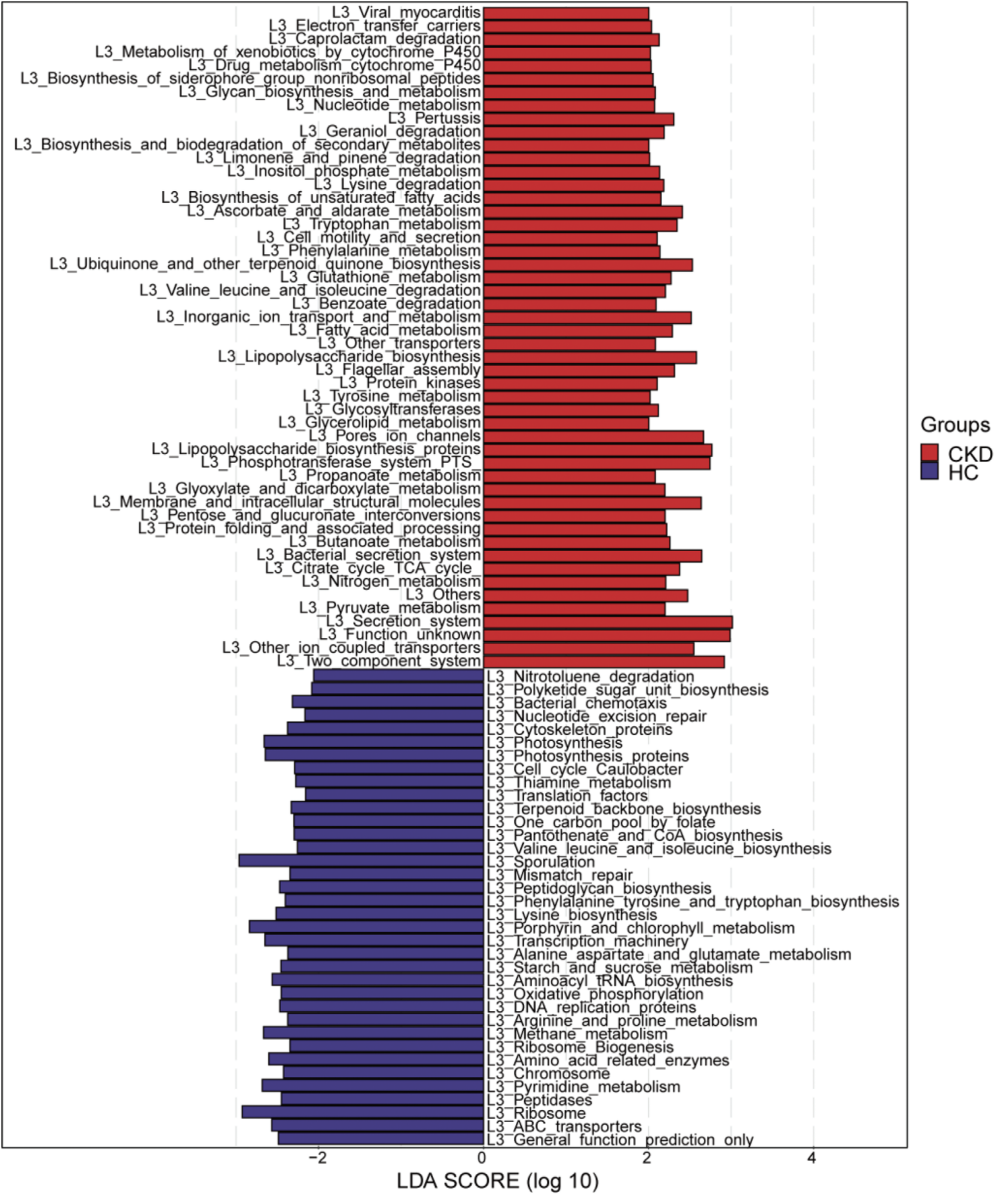
Crucial microbial predicted functions related to CKD. Based on the LDA selection, 50 predicted microbial functions were remarkably increased, while 36 functions were remarkably decreased in CKD (*n* = 110) compared with HC (*n* = 210) (all *p* < 0.05). CKD, chronic kidney disease; HC, healthy controls; LDA, linear discriminant analysis.

The KEGG orthology (KO) and the KEGG pathway/module profile were constructed using the PICRUSt version 1.0.0 pipeline^[^ ^12^ ^]^ and human version 0.99,^[^ ^13^ ^]^ and the 16S rRNA marker gene sequences were used to predict the microbial community function profiles. The gut microbial community function profiles and the predominant microbial functions within CKD and the healthy controls have been shown by a cladogram (Figure S19, supporting Information). Based on the LDA selection, 50 predicted microbial functions including ascorbate and aldarate metabolism, tryptophan metabolism, and phenylalanine metabolism were remarkably increased, while 36 functions including arginine and proline metabolism, starch and sucrose metabolism, and lysine biosynthesis were remarkably decreased in CKD compared with those in the healthy controls (all *p* < 0.05, **Figure** **5**, online supplementary data S9).

### 2.5 Diagnostic Potential of CKD Based on the Gut Microbial Markers

**Figure 6:**
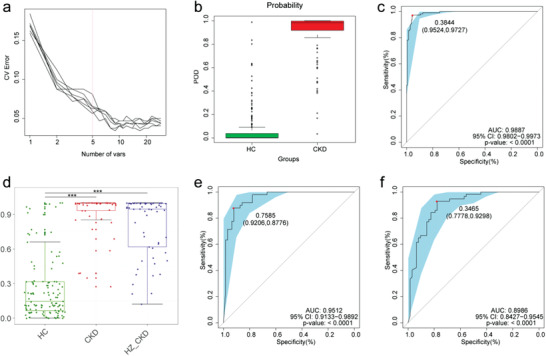
Diagnostic potential of gut microbial markers in CKD patients. a) Five microbial markers were selected as the optimal markers set by the random forest model. b) The POD value was significantly increased in CKD (*n* = 110) versus HC (*n* = 210) in the discovery cohort. c) The POD index achieved an AUC value of 0.9887 with 95% CI of 0.9802 to 0.9973 between CKD (*n* = 110) versus HC (*n* = 210) in the discovery cohort (*p* < 0.0001). d) The POD values were remarkably increased in CKD (*n* = 49) and HZ_CKD (*n* = 57) compared with HC (all *p* < 0.001). e) The POD index achieved an AUC value of 0.9512 with 95% CI of 0.9133 to 0.9892 between CKD (*n* = 49) versus HC (*n* = 63) in the validation cohort (*p* < 0.0001). f) The POD index achieved an AUC value of 0.8986 with 95% CI of 0.8427 to 0.9545 between HZ_CKD (*n* = 57) versus HC (*n* = 63) in the independent diagnostic cohort (*p* < 0.0001). *, *p* < 0.05, **, *p* < 0.01, ***, *p* < 0.001. CV Error, the cross‐validation error; CKD, chronic kidney disease; HZ_CKD, the patients of CKD come from Hangzhou; HC, healthy controls; POD, probability of disease; CI, confidence interval; AUC, area under the curve.

In the discovery cohort, a random forest classifier model between 110 CKD and 210 healthy controls was constructed to assess the potential of gut microbial markers as a non‐invasive diagnostic tool for CKD. Five OTUs were selected as the optimal marker set of CKD by a fivefold cross‐validation of the random forest model (**Figure** **6**a). The relative abundance of the 5 OTUs markers in each sample is shown in the online supplementary data S10. The probability of disease (POD) index of the discovery cohort, the validation cohort, and the independent diagnosis cohort was calculated using the identified optimal 5 OTUs set (online supplementary data S11, S13, and S15, respectively). In the discovery cohort, the POD value of CKD increased significantly compared with that in the healthy controls (*p* < 0.05, Figure 6b), and the POD index reached an area under the receiver operating characteristic (ROC) curve (AUC) of 0.9887 with 95% confidence interval (CI) of 0.9802–0.9973 between the CKD and healthy controls (*p* < 0.0001, Figure 6c). The data indicated that the classifier model based on microbial markers reached a powerful diagnostic potential in distinguishing CKD from healthy controls.

Furthermore, 63 healthy control samples from Zhengzhou were combined with 49 CKD samples from Zhengzhou and 57 CKD samples from Hangzhou, respectively, to form a validation cohort and an independent diagnostic cohort to validate the diagnostic effectiveness of the classifier model for CKD. The relative abundance of the 5 OTUs markers in each sample at the validation cohort and the independent diagnosis cohort is shown in the online supplementary data S12 & S14, respectively. The data showed that the POD values of CKD in both cohorts were remarkably higher than those in healthy controls (all *p* < 0.001, Figure 6d). In the validation cohort, the POD index reached an are under the curve (AUC) value of 0.9512 with a 95% CI of 0.9133–0.9892 between CKD and healthy controls (*p* < 0.0001, Figure 6e). Moreover, the POD index reached an AUC value of 0.8986 with a 95% CI of 0.8427–0.9545 between CKD and healthy controls in the independent diagnostic cohort (*p* < 0.0001, Figure 6f). These data validated a significant diagnostic potential of gut microbial markers for CKD.

### 2.6 Alterations of the Gut Microbiome in Different Clinical Stages of CKD

**Figure 7:**
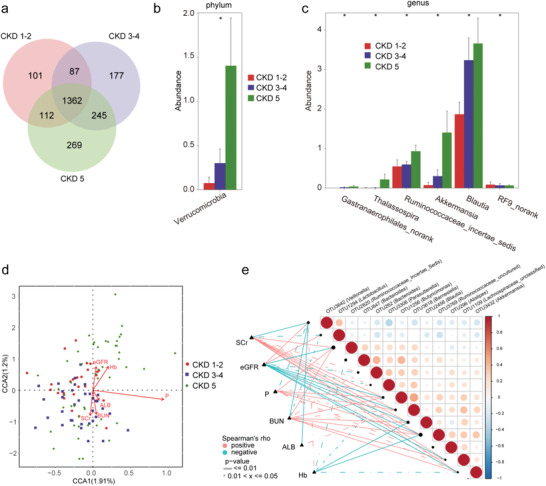
Alterations of the gut microbiome along with the CKD progression. a) A Venn diagram displaying the overlaps among groups showed that the total number of OTUs was 2353, and 1362 OUTs were shared in all groups. Noteworthy, 101 OTUs, 177 OTUs, and 269 OTUs were unique for CKD stages 1–2 (*n* = 26), CKD stages 3–4 (*n* = 36), and CKD stage 5 (*n* = 48), respectively. b) With the progress of CKD, the increased microbial community at the phylum level (*p* < 0.05). c) With the progression of CKD, the increased and decreased microbial community at the genus level (all *p* < 0.05). d) The CCA analysis of the associations between the gut microbiome and clinical indicators for CKD from CCA1 and CCA2 (1.91% and 1.2%). e) Heatmap showing the partial Spearman's correlation coefficients among 13 OTUs and 6 clinical indicators of CKD (*n* = 110). Distance correlation plots of relative abundances of 13 OTUs and the clinical indices SCr, eGFR, P, BUN, ALB, and Hb. CKD, chronic kidney disease; CCA, canonical correspondence analysis; OTUs, operational taxonomy units; Hb, hemoglobin; ALB, albumin; BUN, blood urea nitrogen; SCr, serum creatinine; eGFR, estimated glomerular filtration rate; P, phosphate.

To clarify the alterations of the gut microbiome in different clinical stages of CKD, the 110 samples of CKD in the discovery cohort were divided into three groups according to the clinical stages of CKD. It included 26 samples of CKD stages 1–2 (Group A), 36 samples of CKD stages 3–4 (Group B), and 48 samples of CKD stage 5 (Group C). We characterized and compared the gut microbiome among the three groups. AS estimated by the alpha diversity index, there was no significant difference in gut microbial diversity among the three groups (Figure S20, Supporting Information, online supplementary data S16). A Venn diagram showed that the total abundance of OTUs was 2353, and 1362 OTUs were shared in all groups (**Figure** **7**a). It is worth noting that 101 OTUs, 177 OTUs, and 269 OTUs were unique for CKD stages 1–2, CKD stages 3–4, and CKD stage 5, respectively. At the phylum level, *Verrucomicrobia* was significantly enriched as CKD progressed within the three groups (*p* < 0.05, Figure 7b, online supplementary data S17). Correspondingly, five genera including *Thalassospira*, *Akkermansia*, and *Blautia* were significantly enriched, and the genus *RF9_norank* was significantly reduced as CKD progressed within the three groups (all *p* < 0.05, Figure 7c, online supplementary data S18). Based on the LDA selection, ten microbial taxa including *Akkermansia*, *Blautia*, and *Verrucomicrobia* were enriched in CKD stage 5, *Parasutterella* was enriched in CKD stages 3–4, and *Tenericutes* and *Mollicutes* were enriched in CKD stages 1–2 (*p* < 0.05, Figure S21, Supporting Information, online supplementary data S19).

### 2.7 Correlation between the Gut Microbiome and Clinical Indicators of CKD

We further analyzed the correlations between the gut microbiome and clinical indicators of CKD and found six clinical indicators (eGFR, hemoglobin, SCr, BUN, and ALB) were closely related to the gut microbiome of CKD. Canonical correspondence analysis (CCA) of the CKD gut microbiome and these clinical indicators has been shown in Figure 7d (online supplementary data S20). Furthermore, we analyzed the correlation between 13 OTUs and the six clinical indicators of CKD based on the Spearman correlation analysis (Figure 7e, online supplementary data S21). SCr and BUN were positively correlated with 7 OTUs including OTU2456 (*Blautia*), OTU1256 (*Butyricimonas*), and OTU3432 (*Akkermansia*), while negatively correlated with OTU3642 (*Veillonella*) and OTU1294 (*Lactobacillus*). Moreover, ALB was positively correlated with OTU3618 (*Barnesiella*) and negatively correlated with OTU3642 (*Veillonella*).

## 3 Discussion and Conclusion

Disturbances of the normal gut microbiome have been recognized in the pathogenesis of diverse chronic diseases, such as obesity,^[^ ^14^ ^]^ diabetes,^[^ ^9^ ^]^ and liver cirrhosis.^[^ ^15^ ^]^ In recent years, the role of the gut microbiome in CKD has been gradually explored. For example, the PCS level of gut‐derived uremia toxin increased with the decrease of eGFR,^[^ ^6a^ ^]^ and its baseline concentration has been reported as an independent predictor for cardiovascular events. Another gut‐derived uremia toxin, TMAO, can also induce atherosclerosis syndrome and increase the risk of cardiovascular disease and death in ESRD patients.^[^ ^16^ ^]^ These studies have shown a strong association between the gut microbiome and CKD. However, specific alterations of the gut microbiome in humans with non‐dialysis CKD have been rarely reported.

Our study comprehensively elucidated the gut microbial profiling in non‐dialysis CKD through the Miseq sequencing of a large number of Chinese samples. Noteworthy, we further elucidated the gut microbiome of different clinical stages of CKD and identified the gut microflora associated with the progression of CKD. At the same time, five optimal microbial markers for CKD were identified by the random forest model. The microbial marker‐based CKD classifiers achieved a strong diagnostic potential in distinguishing CKD from healthy controls. More significantly, the CKD classifier also successfully implemented a cross‐regional validation. Regional, dietary, and population genetic factors are the main influencing factors for gut microbiome variation.^[^ ^17^ ^]^ Studies have shown that non‐genetic factors and genetic factors each account for about 10% of the variation of gut flora, and regional variation limits the application of gut microbial disease models to some extent.^[^ ^17^, ^18^ ^]^ Our study is the first to realize cross‐regional verification of the CKD classifier model based on gut microbial markers, which reduces the influence of these variation factors to some extent. These results indicated that targeted biomarkers of the gut microbiome have the potential to be used as a non‐invasive diagnostic tool for CKD. This new diagnostic tool can be used as a supplement to the traditional CKD diagnostic method.

We found that the gut microbial diversity of CKD was significantly reduced compared with that of healthy controls, and the microbial community was significantly different from that of healthy controls. The result indicated that the human gut microbiome changed significantly from a healthy state to the development of CKD. Compared with healthy controls, the abundance of 5 phyla and 36 genera were significantly increased in CKD. This suggests that it is these populations with significant differences in abundance that cause significant alterations in the composition of gut microbiome of non‐dialysis CKD. Meanwhile, 24 genera were dominant in CKD. Among them, *Enterobacteriaceae* possessing urease, indole, and p‐cresol‐forming enzymes, while *Haemophilus and Klebsiella* producing lipopolysaccharide (LPS), IS, and PCS were the main uretic toxins and can be used as indicators of CKD progression.^[^ ^6^, ^7^, ^19^ ^]^ Overgrowth of the gut microbial taxa possessing these enzymes may facilitate the progression of CKD by affecting the synthesis of uremia toxin molecules associated with the progression of CKD. In addition, high levels of LPS activate the NF‐KB pathway and promote the production of pro‐inflammatory cytokines (IL‐1, IL‐6, and TNF‐*α*), leading to systemic inflammation and the progression of CKD.^[^ ^20^ ^]^

Different microbial productions and functions contribute to the pathogenesis and development of different diseases.^[^ ^21^ ^]^ In our study, the metabolism of ascorbate and aromatic amino acids (phenylalanine, tryptophan) and LPS biosynthesis were significantly increased in CKD, while the metabolism of arginine and proline was significantly decreased. Catabolism of ascorbic acid can produce oxalic acid (OA),^[^ ^22^ ^]^ increased metabolism of tryptophan can promote the production of indoles,^[^ ^20^, ^23^ ^]^ and enhanced metabolism of phenylalanine contributes to the production of para‐cresol. Indoles and para‐cresol are the precursor substances for the synthesis of uremia toxins related to the progression of CKD, while excessive elevation of OA can damage renal function,^[^ ^20^, ^23^ ^]^ and high levels of LPSs can also promote systemic inflammatory reactions leading to the progression of CKD.^[^ ^20^ ^]^ This suggests that the alterations in gut microbiome function in non‐dialysis CKD also contribute to the production of metabolites associated with the progression of CKD. In addition, it was found that butanoate metabolism and biosynthesis of siderophore group nonribosomal peptides increased significantly in CKD gut microbial prediction function. Currently, no studies have been able to confirm the correlation between these functional alterations and CKD, but their significant alterations in the gut microbiome of CKD suggest that they may have important physiological significance n the occurrence and development of CKD, which is a research direction worthy of further exploration.

We further analyzed and compared the gut microbiome of non‐dialysis CKD at different clinical stages, and found that the abundance of *Thalassospira*, *Akkermansia* and *Ruminococcaceae incertae sedis* increased along with the progression of CKD, suggesting that these gut microbiota play an important role in the disease progression of CKD and may be the key pathogenic bacteria causing the progression of CKD. Studies by Wang et al.^[^ ^24^ ^]^ have shown that in addition to renal function, gut flora appears to be an important determinant of host fecal and serum metabolic landscapes, and that species associated with uremia toxin production are directly and closely related to ESRD clinical variables. We further analyzed the correlation between clinical indicators reflecting the severity of CKD disease and gut microbiome, and found that the abundance of *Akkermansia* was positively correlated with SCr and BUN levels, and negatively correlated with eGFR and hemoglobin levels. This suggests that *Akkermansia* plays a crucial role in the progression of CKD and may be a microbial species closely related to the production of uremia toxins. This finding is of great significance and can provide a direction for further exploration of new therapeutic targets for CKD based on gut microbiome.

In conclusion, our study demonstrated the gut microbiome characteristics of non‐dialysis CKD in a large clinical cohort, identified specific microbial markers, and demonstrated the potential of microbial markers as a non‐invasive diagnostic tool for CKD. At the same time, we explained the alterations of the gut microbiome in CKD of different clinical stages and identified the gut microbiota associated with the progression of CKD. These are the advantages of this study. However, the progression of CKD is closely related to the metabolites of gut microbiome. Our study lacks the detection of gut microbial metabolites in patients with CKD, which is the deficiency of this study. Nevertheless, our data provide a comprehensive investigation of the gut microbiome of patients with CKD from a large cohort sample and raises the possibility of using non‐invasive biomarkers to diagnose CKD, providing new insights into the association between CKD and gut microbiome.

## 4 Experimental Section

##### Participant Information

This study was designed based on the principle of the PRoBE design (prospective specimen collection and retrospective blinded evaluation).^[^ ^25^ ^]^ The study was conducted in accordance with the Declaration of Helsinki and the Rules of Good Clinical Practice. The whole study was reviewed and approved by the Institutional Review Board of the First Affiliated Hospital of Zhengzhou University. Informed consents on enrolment had been signed and provided by all participants. Demographics and clinical data of the participants were obtained from questionnaires and hospital electronic medical records of hospitals.

##### Inclusion and Exclusion Criteria

The diagnosis and staging of all patients with CKD were in accordance with the diagnostic criteria and staging criteria for CKD in the KDIGO 2012 clinical practice guideline for the evaluation and management of CKD.^[^ ^26^ ^]^ Exclusion criteria were as follows: a) antibiotics or probiotics used in the past 4 weeks; b) CKD‐related drug therapy had been initiated or hemodialysis or peritoneal dialysis had been performed; c) presence of other diseases such as liver disease, digestive disease, diabetes, and tumor; and d) participants missing clinical information.

The control group consisted of 300 healthy volunteers who visited the hospital for their annual physical examination. They had to fulfil the following inclusion criteria in order to be included in the experiment: a) hemoglobin, liver function, kidney function, electrolytes, urine, and stool were normal; b) the absence of hypertension, diabetes, obesity, liver disease, digestive disease, and tumor; c) the absence of the hepatitis B/C virus antigen; and d) did not take antibiotics and/or probiotics within 4 weeks before sample collection.

##### Fecal sample Collection and DNA Extraction

A fresh fecal sample of each participant was provided between 06:00 and 08:00 a.m. Routine fecal testing was performed on each fecal sample. Each sample was divided into five equal parts of 200 mg and was frozen at −80 °C immediately. The DNA extraction was conducted as per a method described previously.^[^ ^27^ ^]^ Briefly, the bacterial genomic DNA was extracted by the Quick gel extraction kit (Qiagen, Germany). NanoDrop (Thermo Scientific) was used to measure DNA concentration and agarose gel electrophoresis was used to estimate the molecular size.

##### PCR Amplification, Miseq Sequencing, and Sequence Data Process

A set of primers targeting the high‐variant v3–v4 region (338F/806R) of the 16S rRNA gene were used to amplify the extracted DNA samples by PCR. PCR products were detected on a 2% w/v agarose gel, and the band was extracted and purified using the AxyPrepDNA Gel (Axygen, CA, USA) and the PCR Clean‐up System. The purified PCR product for each sample was mixed. The DNA library was constructed according to the manufacturer's instructions. Sequencing was performed on an Illumina MiSeq platform by Shanghai Mobio Biomedical Technology Co., Ltd., China. The raw Illumina read data for all samples were deposited in the European Bioinformatics Institute European Nucleotide Archive database under the accession number PRJNA562327. The amplicon reads were processed by the following steps: a) FLASH version 1.2.10^[^ ^28^ ^]^ with default parameters overlapped the pair‐end sequenced reads of each library; b) A custom per program was used to perform more specific quality control of overlapped reads generated by FLASH: 1) Ambiguous bases (N) were not allowed in reads, 2) No more than five mismatches were allowed in overlap region, 3) Mismatches were not allowed in the barcode primer region; c) Reads were de‐multiplexed and assigned into different samples based on the barcodes; d) Chimeric sequences were detected and removed with UCHIME version 4.2.40,^[^ ^29^ ^]^ and the 16S “golden standard” database provided by Broad Institute (version microbiome util‐r20110519, http://drive5.com/uchime/gold.fa) was used as a reference to match the OTUs.

##### OTUs Clustering and Taxonomy Annotation

Reads were randomly selected from all samples of the equal number, and then OTUs were binned using the UPARSE pipeline^[^ ^30^ ^]^ through the following steps: a) deleted the abundant sequences and singletons first; b) used the command “usearch‐cluster_OTUs” to bin the unique sequences into OTUs; c) used the command “usearch‐usearch_global‐id 0.97” to align against the randomly selected sequences with the OTU sequences, set the identity threshold as 0.97, and then created an OTUs composition table. All OTUs for the samples in the discovery cohort, validation cohort, and independent diagnosis cohort were collected. The sequences were annotated with the RDP classifier version 2.6^[^ ^31^ ^]^ and set the confidence level as 0.5 according to the developer's documents (http://rdp.cme.msu.edu/classifier/class_help.jsp#conf).

##### Bacterial Diversity and Taxonomic Analysis

By a sampling‐based OTUs analysis, bacterial diversity was determined and shown by the Shannon index, the Chao index, and the Ace index, which used the R program package “vegan” for calculations.^[^ ^32^ ^]^ NMDS, PCA, and PCoA were conducted by the R package (http://www.R-project.org/) to display the microbiome space between both the group samples. A heatmap that identified key variables was accomplished by the heatmap builder.

Bacterial taxonomic analyses and comparison between both groups by the Wilcoxon rank‐sum test were conducted, which included the bacterial phylum and genus. Based on the normalized relative abundance matrix, the LEfSe method (http://huttenhower.sph.harvard.edu/lefse/) was applied to analyze fecal microbial characterization between cases and healthy controls.^[^ ^33^ ^]^ This method first used the Kruskal–Wallis rank‐sum test (*p* < 0.05) to detect features with a significant differential abundance, then evaluated the effect size of each feature by LDA (LDA score (log10) = 2 as cut‐off value).^[^ ^34^ ^]^

##### Functional Annotation of 16S rRNA Gene Based on the KEGG Profile

The KEGG orthology (KO) and the KEGG pathway/module profile were constructed by the PICRUSt version 1.0.0 pipeline^[^ ^12^ ^]^ and human version 0.99,^[^ ^13^ ^]^ and 16S rRNA marker gene sequences were used to predict the microbial community function profiles. PICRUSt recaptures key findings from the Human Microbiome Project by an extended ancestral‐state reconstruction algorithm and accurately predicts abundance in host‐associated communities of the gene families, with a quantifiable uncertainty.

##### Identification of the OTU Biomarker and Construction of POD

The discovery OTU frequency profile and the validation OTU frequency profile by mapping reads from the discovery cohort and the validation cohort against these represented sequences, respectively, were obtained. The Wilcoxon test was used to determine the significance (*p* < 0.05), based on which 47 OTUs were selected for further analysis. The 47 OTUs abundance profile of the discovery cohort were used for fivefold cross‐validation. The verification was performed on a random forest model, in which all parameters were default except for “importance = TRUE.” Then the cross‐validation error curve was obtained by using five trials of the fivefold cross‐validation. The point with the minimum cross‐validation error as the cut‐off point was taken, and the minimum error plus the SD of the corresponding point was used to determine the cut‐off value. All sets of OTU markers with the error less than the cut‐off value were selected, and the set with the smallest number of OTUs was considered as the optimal set. Finally, the determined optimal OTUs set was used to calculate the POD index of the discovery cohort and the verification cohort. The POD index as the ratio of the number of randomly generated decision trees that predicted the sample as “CKD” and the number that predicted healthy controls was defined. The ROC curve was constructed (R 3.3.0, pROC package) to evaluate the constructed models, and the AUC was used to represent the ROC effect.

##### Statistical Analysis

Continuous variables were presented with the form of means (standard deviations) or median (interquartile ranges). Categorical variables were presented with the form of percentages. Differences between subjects in CKD (*n* = 110, *n* = 49) and healthy controls (*n* = 210, *n* = 63) were compared by using Student's *t*‐test for normal continuous variables, Wilcoxon rank‐sum test for non‐normal continuous variables, and Chi‐square test or Fisher's exact test for categorical variables. Statistical analyses were performed using the SPSS V.21.0 for Windows (SPSS, Chicago, Illinois, USA). Statistical significance was defined by *p* < 0.05 (two tailed), without post‐analysis and *α* adjustment. Data transformation, normalization, evaluation of outliers was not used in the study.

## Data Availability

The raw Illumina read data for all samples were deposited in the European Bioinformatics Institute European Nucleotide Archive database under the accession number PRJNA562327.

## Conflict of Interest

The authors declare no conflict of interest.

## Author Contributions

Z.R., Y.F., A.L., and Q.S. contributed equally to this work. G.W., Z.L., Z.Y., and L.L. designed the study. Z.R., Y.F., Q.S., L.R., S.D., W.L., D.G., and S.G. collected clinical samples. H.L. and Z.W. extracted the bacterial DNA. A.L., J.W., H.R., and C.L. analyzed the data. Z.R., Y.F., and G.W. wrote the manuscript. All authors reviewed and approved the manuscript.

## Supporting information

Supporting Information

Supporting pdf

Supporting Excel 1

Supporting Excel 2

Supporting Excel 3

Supporting Excel 4

Supporting Excel 5

Supporting Excel 6

Supporting Excel 7

Supporting Excel 8

Supporting Excel 9

Supporting Excel 10

Supporting Excel 11

Supporting Excel 12

Supporting Excel 13

Supporting Excel 14

Supporting Excel 15

Supporting Excel 16

Supporting Excel 17

Supporting Excel 18

Supporting Excel 19

Supporting Excel 20

Supporting Excel 21
