# Supplementary material for: Alterations of the Human Gut Microbiome in Chronic Kidney Disease
Source: Adv Sci (Weinh). 2020 Sep 2;7(20):2001936. doi: 10.1002/advs.202001936 (PMC7578882; doi:10.1002/advs.202001936)
Supplement: Supplementary file 1 — Supporting Information [file ADVS-7-2001936-s001.pdf]

© 2020 Wiley-VCH GmbH

ADVANCED SCIENCE
Open Access

Supporting Information

for *Adv. Sci.*, DOI: 10.1002/advs. 202001936

Alterations of the Human Gut Microbiome in Chronic Kidney Disease

Zhigang Ren, Yajuan Fan, Ang Li, Quanquan Shen, Jian Wu, Lingyan Ren, Haifeng Lu, Suying Ding, Hongyan Ren, Chao Liu, Wenli Liu, Dan Gao, Zhongwen Wu, Shiyuan Guo, Ge Wu, Zhangsuo Liu,* Zujiang Yu,* and Lanjuan Li**

Supplementary data legends

Alterations of the human gut microbiome in chronic kidney disease

Authors

Zhigang Ren[1, 10†], Yajuan Fan[2†], Ang Li[1, 7, 10†], Quanquan Shen[3, 11†], Jian Wu[4], Lingyan Ren[5], Haifeng Lu[6], Suying Ding[7], Hongyan Ren[8], Chao Liu[8], Wenli Liu[9], Dan Gao[2], Zhongwen Wu[6], Shiyuan Guo[2], Ge Wu[2*], Zhangsuo Liu[2*], Zujiang Yu[1, 10*], and Lanjuan Li[6*]

Affiliations

[1] Department of Infectious Diseases, the First Affiliated Hospital of Zhengzhou University, Zhengzhou 450052, China.

[2] Department of Nephrology, the First Affiliated Hospital of Zhengzhou University, Zhengzhou 450052, China.

[3] Department of Nephrology, Zhejiang Provincial People's Hospital, People's Hospital of Hangzhou Medical College, Hangzhou, Zhejiang 310014, China.

[4] College of Public Health, Zhengzhou University, Zhengzhou 450052, China.

[5] Department of Nephrology, the First Affiliated Hospital of Huzhou Teachers College, the First People's Hospital of Huzhou, Huzhou, Zhejiang 313000, China.

[6] State Key Laboratory for Diagnosis and Treatment of Infectious Disease; National Clinical Research Center for Infectious Diseases; Collaborative Innovation Center for Diagnosis and Treatment of Infectious Diseases, the First Affiliated Hospital, School of Medicine, Zhejiang University, Hangzhou 310003, China.

[7] Health Management Center, the First Affiliated Hospital of Zhengzhou University, Zhengzhou 450052, China.

[8] Shanghai Mobio Biomedical Technology Co., Ltd., Shanghai 201111, China.

[9] Clinical Laboratory Diagnostics, Medical Technology College, Beihua University, Jilin 132013, China.

[10] Gene Hospital of Henan Province; Precision Medicine Center, the First Affiliated Hospital of Zhengzhou University, Zhengzhou 450052, China.

[11] Department of Nephrology, Chun'an First People's Hospital, Hangzhou, Zhejiang, China.

† These authors contributed equally to this work.

***Corresponding to**

Ge Wu, Prof., M.D., Department of Nephrology, the First Affiliated Hospital of Zhengzhou University, No. 1, Jianshe East Road, Zhengzhou 450052, China. E-mail: fccwug@zzu.edu.cn.

Zhangsuo Liu, Ph.D, M.D., Department of Nephrology, the First Affiliated Hospital of Zhengzhou University, No. 1, Jianshe East Road, Zhengzhou 450052, China.
E-mail: zhangsuoliu@zzu.edu.cn.

Zujiang Yu, Ph.D, M.D., Department of Infectious Diseases, the First Affiliated Hospital of Zhengzhou University, Zhengzhou 450052, China. E-mail: johnyuem@zzu.edu.cn.

Lanjuan Li, Prof., M.D., State Key Laboratory for Diagnosis and Treatment of Infectious Disease, the First Affiliated Hospital, School of Medicine, Zhejiang University, #79 Qingchun Road, Hangzhou 310003, China. E-mail: ljli@zju.edu.cn.

Supplementary data S1-21:

Supplementary data S1. Clinic data of all Chinese individuals in the discovery cohort (n=320), validation cohort (n=112) and independent diagnosis cohort (n=57).

Supplementary data S2. The detailed values of gut microbial diversity index and observed OTUs in the discovery cohort (210 healthy controls and 110 CKD).

Supplementary data S3. The relative abundance and distribution of the key 47 OTUs in the discovery cohort (210 healthy controls and 110 CKD).

Supplementary data S4. The abundance and composition at the phylum level of each

sample in the discovery cohort (210 healthy controls and 110 CKD).

Supplementary data S5. The different degree of phylum level (p value) between the healthy controls (n=210) and CKD (n=110) in the discovery cohort.

Supplementary data S6. The abundance and composition of each sample in the discovery cohort (210 healthy controls and 110 CKD) at the genus level.

Supplementary data S7. The different degree of genus level (p value) between the healthy controls (n=210) and CKD (n=110) in the discovery cohort.

Supplementary data S8. The corresponding LDA value and p value of the bio-makers in the discovery cohort (210 healthy controls and 110 CKD).

Supplementary data S9. The corresponding LDA value and p value of microbial community gene function for samples in the discovery cohort (210 healthy controls and 110 CKD).

Supplementary data S10. By random forest classifier model, the corresponding output value of each optimal microbial marker in the discovery cohort (210 healthy controls and 110 CKD).

Supplementary data S11. The corresponding POD value for each sample in the discovery cohort (210 healthy controls and 110 CKD).

Supplementary data S12. By random forest classifier model, the corresponding output value of each optimal microbial marker in the validation cohort (63 healthy controls and 49 CKD).

Supplementary data S13. The corresponding POD value for each sample in validation cohort (63 healthy controls and 49 CKD).

Supplementary data S14. By random forest classifier model, the corresponding output value of each optimal microbial marker in the independent diagnosis cohort (57 CKD from Hangzhou, china and 63 healthy controls from Zhengzhou, china).

Supplementary data S15. The corresponding POD value of each sample in the independent diagnosis cohort (57 CKD from Hangzhou, china and 63 healthy controls from Zhengzhou, china).

Supplementary data S16. The detailed values of gut microbial diversity index and

observed OTUs in Group A (26 patients of CKD 1-2 stage), Group B (36 patients of CKD 3-4 stage) and Group C (48 patients of CKD 5 stage).

Supplementary data S17. The different degree of phylum level (p value) among the Group A (26 patients of CKD 1-2 stage), Group B (36 patients of CKD 3-4 stage) and Group C (48 patients of CKD 5 stage).

Supplementary data S18. The different degree of genus level (p value) among the Group A (26 patients of CKD 1-2 stage), Group B (36 patients of CKD 3-4 stage) and Group C (48 patients of CKD 5 stage).

Supplementary data S19. The bacterial abundance and composition of each sample in Group A (26 patients of CKD 1-2 stage), Group B (36 patients of CKD 3-4 stage) and Group C (48 patients of CKD 5 stage).

Supplementary data S20. The data of canonical correspondence analysis (CCA) between gut microbiome and clinical indicators of CKD (n=110).

Supplementary data S21. The p value between OTUs and clinical indicators of CKD (n=110) obtained by spearman correlation analysis.
