# Supplementary material for: Alterations of the Human Gut Microbiome in Chronic Kidney Disease
Source: Adv Sci (Weinh). 2020 Sep 2;7(20):2001936. doi: 10.1002/advs.202001936 (PMC7578882; doi:10.1002/advs.202001936)
Supplement: Supplementary file 2 — Supporting pdf [file ADVS-7-2001936-s002.pdf]

© 2020 Wiley-VCH GmbH

ADVANCED SCIENCE
Open Access

Supporting Information

for *Adv. Sci.*, DOI: 10.1002/advs. 202001936

Alterations of the Human Gut Microbiome in Chronic Kidney Disease

Zhigang Ren, Yajuan Fan, Ang Li, Quanquan Shen, Jian Wu, Lingyan Ren, Haifeng Lu, Suying Ding, Hongyan Ren, Chao Liu, Wenli Liu, Dan Gao, Zhongwen Wu, Shiyuan Guo, Ge Wu, Zhangsuo Liu,* Zujiang Yu,* and Lanjuan Li**

Supplementary figures legends

Alterations of the human gut microbiome in chronic kidney disease

Authors

Zhigang Ren[1, 10†], Yajuan Fan[2†], Ang Li[1, 7, 10†], Quanquan Shen[3, 11†], Jian Wu[4], Lingyan Ren[5], Haifeng Lu[6], Suying Ding[7], Hongyan Ren[8], Chao Liu[8], Wenli Liu[9], Dan Gao[2], Zhongwen Wu[6], Shiyuan Guo[2], Ge Wu[2]*, Zhangsuo Liu[2]*, Zujiang Yu[1, 10]*, and Lanjuan Li[6]*

Affiliations

[1] Department of Infectious Diseases, the First Affiliated Hospital of Zhengzhou University, Zhengzhou 450052, China.

[2] Department of Nephrology, the First Affiliated Hospital of Zhengzhou University, Zhengzhou 450052, China.

[3] Department of Nephrology, Zhejiang Provincial People's Hospital, People's Hospital of Hangzhou Medical College, Hangzhou, Zhejiang 310014, China.

[4] College of Public Health, Zhengzhou University, Zhengzhou 450052, China.

[5] Department of Nephrology, the First Affiliated Hospital of Huzhou Teachers College, the First People's Hospital of Huzhou, Huzhou, Zhejiang 313000, China.

[6] State Key Laboratory for Diagnosis and Treatment of Infectious Disease; National Clinical Research Center for Infectious Diseases; Collaborative Innovation Center for Diagnosis and Treatment of Infectious Diseases, the First Affiliated Hospital, School of Medicine, Zhejiang University, Hangzhou 310003, China.

[7] Health Management Center, the First Affiliated Hospital of Zhengzhou University, Zhengzhou 450052, China.

[8] Shanghai Mobio Biomedical Technology Co., Ltd., Shanghai 201111, China.

[9] Clinical Laboratory Diagnostics, Medical Technology College, Beihua University, Jilin 132013, China.

[10] Gene Hospital of Henan Province; Precision Medicine Center, the First Affiliated Hospital of Zhengzhou University, Zhengzhou 450052, China.

[11] Department of Nephrology, Chun'an First People's Hospital, Hangzhou, Zhejiang, China.

[†] These authors contributed equally to this work.

***Corresponding to**

Ge Wu, Prof., M.D., Department of Nephrology, the First Affiliated Hospital of Zhengzhou University, No. 1, Jianshe East Road, Zhengzhou 450052, China. E-mail: fccwug@zzu.edu.cn.

Zhangsuo Liu, Ph.D, M.D., Department of Nephrology, the First Affiliated Hospital of Zhengzhou University, No. 1, Jianshe East Road, Zhengzhou 450052, China.
E-mail: zhangsuoliu@zzu.edu.cn.

Zujiang Yu, Ph.D, M.D., Department of Infectious Diseases, the First Affiliated Hospital of Zhengzhou University, Zhengzhou 450052, China. E-mail: johnyuem@zzu.edu.cn.

Lanjuan Li, Prof., M.D., State Key Laboratory for Diagnosis and Treatment of Infectious Disease, the First Affiliated Hospital, School of Medicine, Zhejiang University, #79 Qingchun Road, Hangzhou 310003, China. E-mail: ljli@zju.edu.cn.

Supplementary figures

1. **Informed consent form and information collection in the study (Translated from Chinese) 4**
2. **Informed consent form for scientific research 5**
3. **Supplementary figures S1-21:**

Supplementary figure S1 8

Supplementary figure S2 9

Supplementary figure S3 9

Supplementary figure S4 10

Supplementary figure S5 10

Supplementary figure S6 11

Supplementary figure S7 12

Supplementary figure S8 13

Supplementary figure S9 14

Supplementary figure S10 15

Supplementary figure S11 16

Supplementary figure S12 17

Supplementary figure S13 18

Supplementary figure S14 19

Supplementary figure S15 20

Supplementary figure S16 21

Supplementary figure S17 22

Supplementary figure S18 23

Supplementary figure S19 24

Supplementary figure S20 25

Supplementary figure S21 26

Informed consent form and information collection
(Translated from Chinese)

We are from Department of Nephrology, the First Affiliated Hospital of Zhengzhou University. We will free of charge help you monitor your gut microbial community, thereby analyzing whether gut microbiota is dysbiosis and the degree of imbalance. These results will provide auxiliary data for clinical diagnosis and treatment. Now, you just provide samples from the stool and tone coat according to our instruction. The whole process keeps free of charge. These results will be used for scientific research. Thank you for your corporation.

Number:<br>Diagnosis:

Patient Sign：

Date:

Patient information collection

Name	Gender	Birth date	Height(cm)	Weight (kg)	BMI	Tel
Floor ward	Admission number	Dietary habit (vegetarian diet/meat/Mixture)	Antibiotics use within 2 months	Yoghourt and probiotics	Previously critical Illness	Long-term drug use
Drinking	Alcohol type	Drinking quantity	Time of Duration	Whether or not abstinence	HBV	Etiology

Informed consent form for scientific research

(Translated from Chinese)

Dear participants,

We are from Department of Nephrology, the First Affiliated Hospital of Zhengzhou University. We will free of charge help you monitor your healthy condition and record your clinical information and healthy/disease status or disease progression process. The collected fecal and urea samples from participants in hospital will be used for scientific research. These results and data from the hospital electronic medical records will provide auxiliary data for clinical diagnosis and treatment, and will be used for scientific research. Thank you for your corporation.

Number:	Diagnosis:

The information that we collect from this research project will be kept confidential. Information about you that will be collected during the research will be put away and no-one but the researchers will be able to see it. Any information about you will have a number on it instead of your name. Only the researchers will know what your number is and we will lock that information up with a lock and key. It will not be shared with or given to anyone except our research team.

The knowledge that we get from doing this research will be shared with you through community meetings before it is made widely available to the public. Confidential information will not be shared. There will be small meetings in the community and these will be announced. After these meetings, we will publish the results in order that other interested people may learn from our research.

I have read the foregoing information, or it has been read to me. I have had the opportunity to ask questions about it and any questions that I have asked have been

answered to my satisfaction. I consent voluntarily to participate as a participant in this research.

Print Name of Participant__________________

Signature of Participant ___________________

Date ___________________________

Day/month/year

A literate witness must sign (if possible, this person should be selected by the participant and should have no connection to the research team). Participants who are illiterate should include their thumb-print as well.

I have witnessed the accurate reading of the consent form to the potential participant, and the individual has had the opportunity to ask questions. I confirm that the individual has given consent freely.

Print name of witness_____________________ AND

Thumb print of participant

Signature of witness ______________________

Date ________________________

Day/month/year

Statement by the researcher/person taking consent

I have accurately read out the information sheet to the potential participant, and to the best of my ability made sure that the participant understands that the following will be done:

1. We will free of charge help you monitor your healthy condition and record your clinical information and healthy/disease status or disease progression process.

2. These data from hospital electronic medical records will be used for scientific research.

3. The collected fecal and urea samples will be used for scientific research.

I confirm that the participant was given an opportunity to ask questions about the study, and all the questions asked by the participant have been answered correctly and to the best of my ability. I confirm that the individual has not been coerced into giving consent, and the consent has been given freely and voluntarily.

A copy of this ICF has been provided to the participant.

Print Name of Researcher/person taking the consent________________________

Signature of Researcher /person taking the consent__________________________

Date ___________________________

Day/month/year

Supplementary Figure

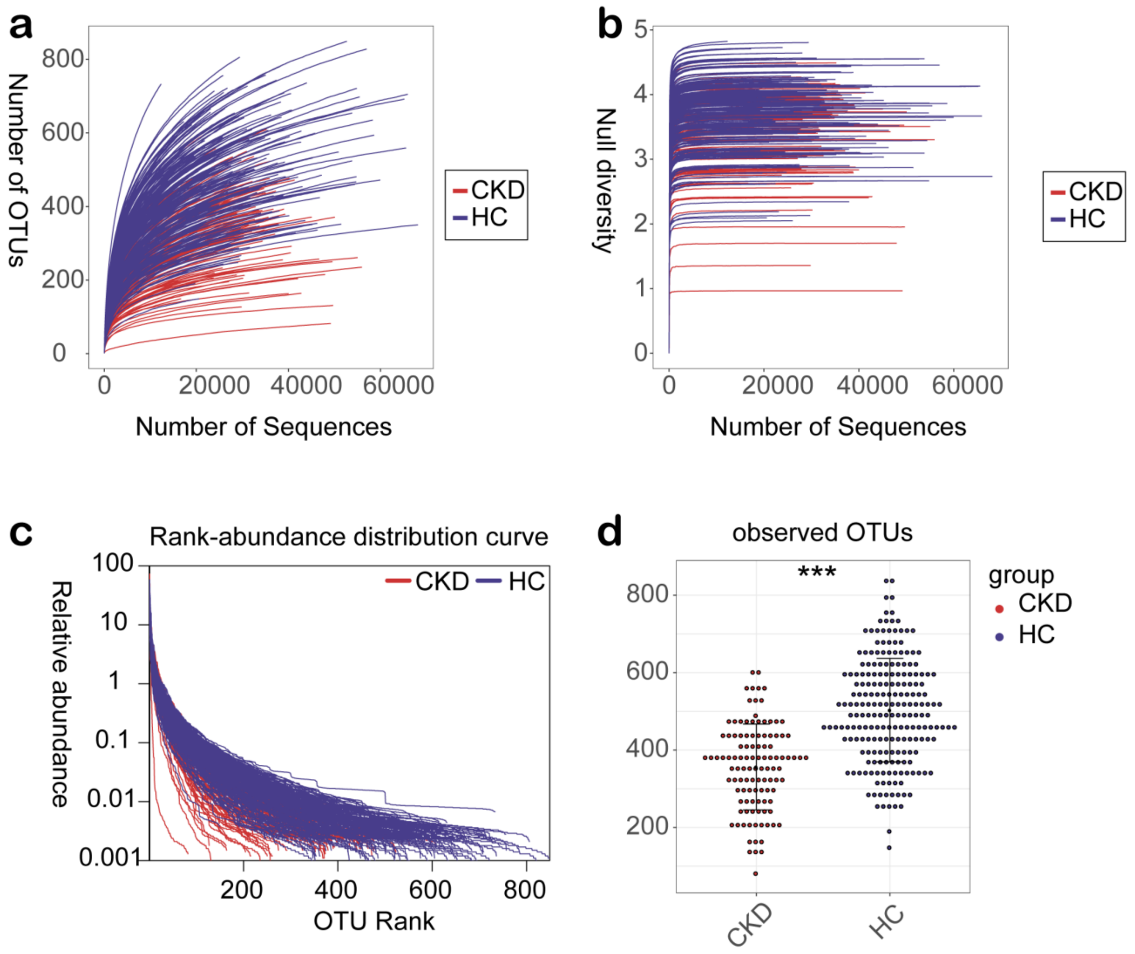

Figure S1. (a) A rarefaction curve between the number of OTUs and the number of sequences in CKD (n=110) and HC (n=210); (b) A shannon-wiener curve between the number of sequences and the null diversity in CKD (n=110) and HC (n=210); (c) A rank-abundance distribution curve for the OTUs of CKD (n=110) and HC (n=210); (d) At the discovery cohort, observed OTUs were significantly decreased in CKD (n=110) versus HC (n=210) (*P*<0.001). ***, *P*<0.001. CKD, chronic kidney disease; HC, healthy controls; OTUs, operational taxonomic units.

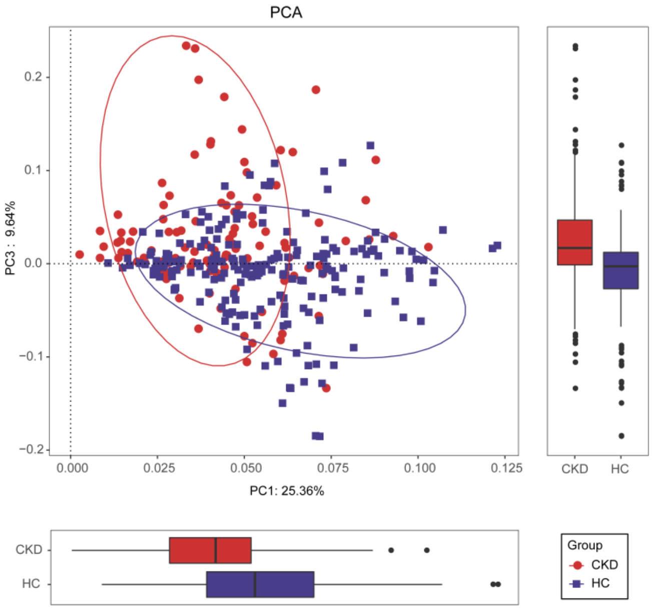

Figure S2. The PCA of gut microbial community for CKD (n=110) and HC (n=210) in the unifrac plot from PC1 and PC3 (25.36% and 9.64%). CKD, chronic kidney disease; HC, healthy controls; PCA, principal component analysis.

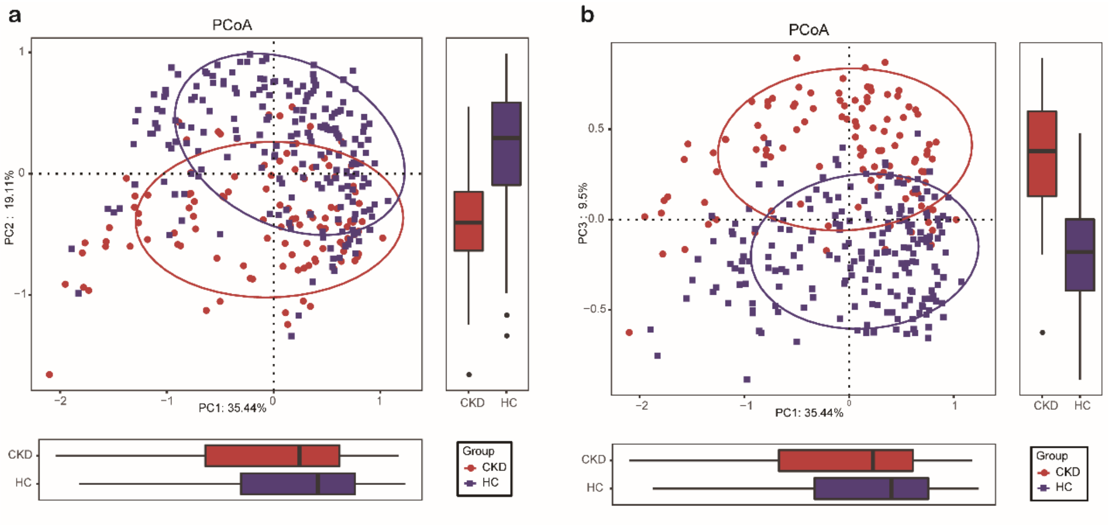

Figure S3. (a) The PCoA of gut microbial community for CKD (n=110) and HC (n=210) in the unweighted Unifrac plot from PC1 and PC2 (35.44% and 19.11%); (b) The PCoA of gut microbial community for CKD (n=110) and HC (n=210) in the unweighted Unifrac plot from PC1 and PC3 (35.44% and 9.5%). CKD, chronic kidney disease; HC, healthy controls; PCoA, principal coordinate analysis.

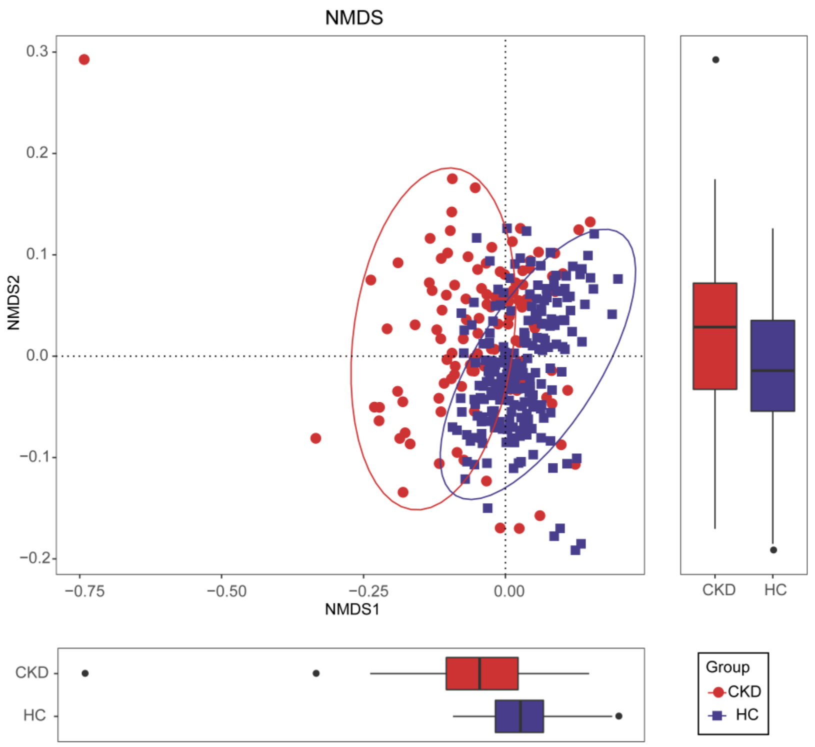

Figure S4. The NMDS analysis of gut microbial community for CKD (n=110) and HC (n=210) in the unweighted Unifrac plot from NMDS1 and NMDS2. CKD, chronic kidney disease; HC, healthy controls; NMDS, non-metric multi-dimensional scaling.

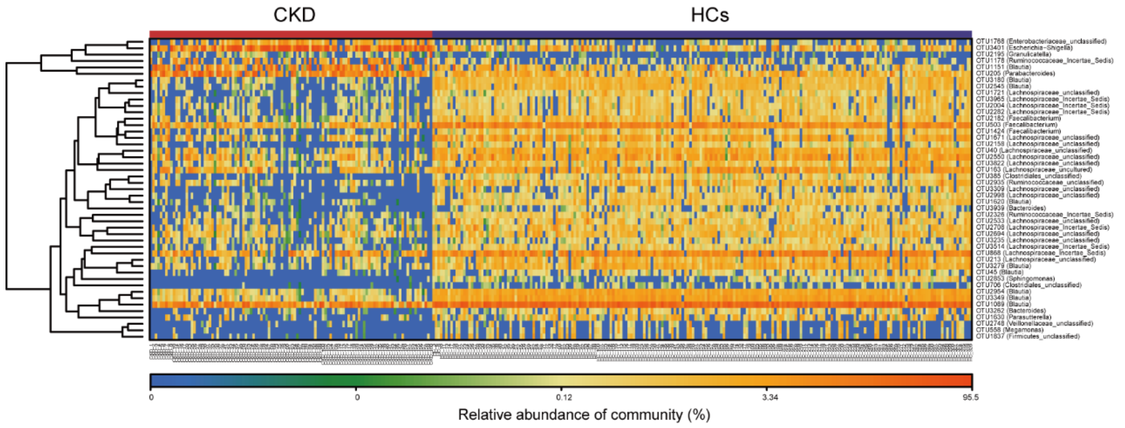

Figure S5. A heatmap showed the relative abundance and distribution of the key 47 OTUs among all samples of the discovery cohort (n=320). Only 6 OTUs were markedly enriched in CKD (n=110), while 41 OTUs were markedly enriched in HC (n=210). CKD, chronic kidney disease; HCs, healthy controls; OTUs, operational taxonomic units.

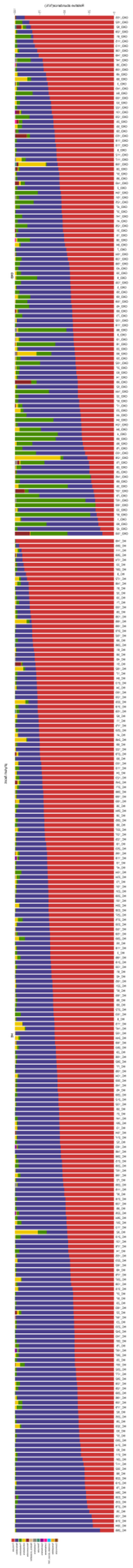

Figure S6. The composition and abundance of bacterial community at the phylum level in each sample of the discovery cohort (n=320). CKD, chronic kidney disease;

HC, healthy controls.

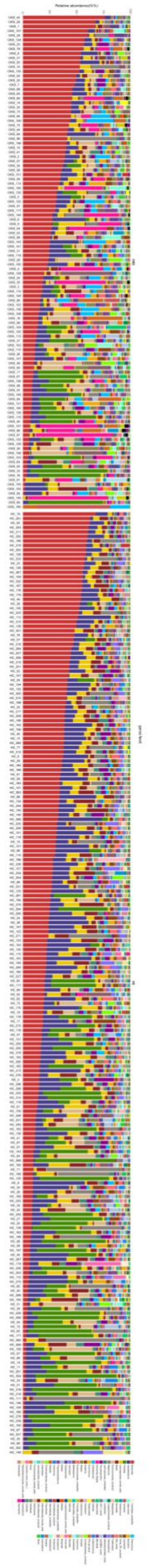

Figure S7. The composition and abundance of bacterial community at the genus level

in each sample of the discovery cohort (n=320). CKD, chronic kidney disease; HC, healthy controls.

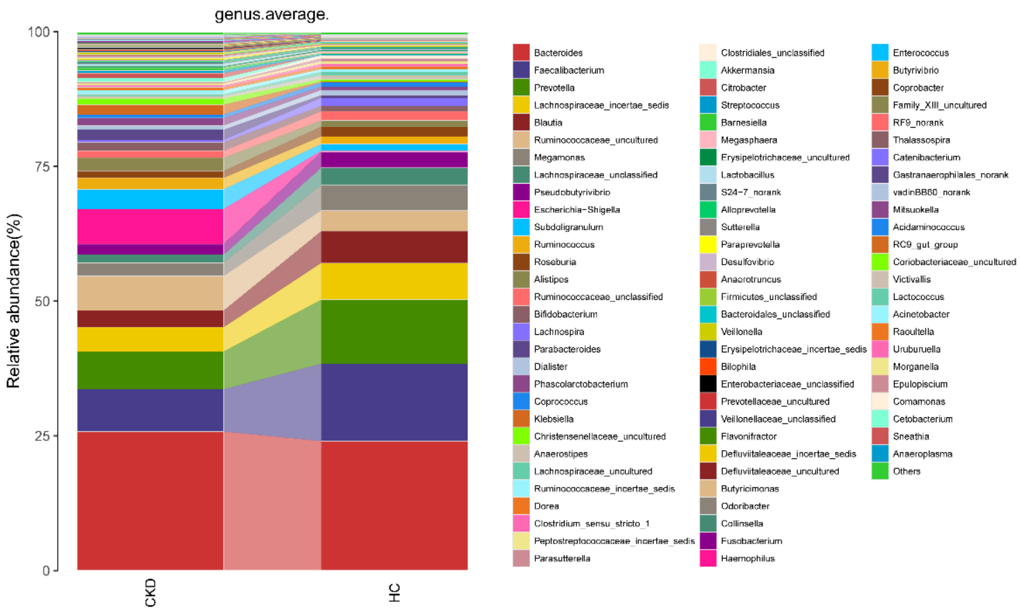

Figure S8. The average composition and relative abundance of the bacterial communities of CKD (n=110) and HC (n=210) at the genus level in the discovery cohort. CKD, chronic kidney disease; HC, healthy controls.

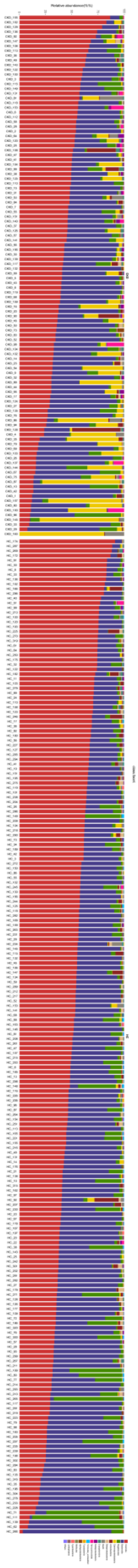

Figure S9. The composition and abundance of bacterial community at the class level in each sample of the discovery cohort (n=320). CKD, chronic kidney disease; HC, healthy controls.

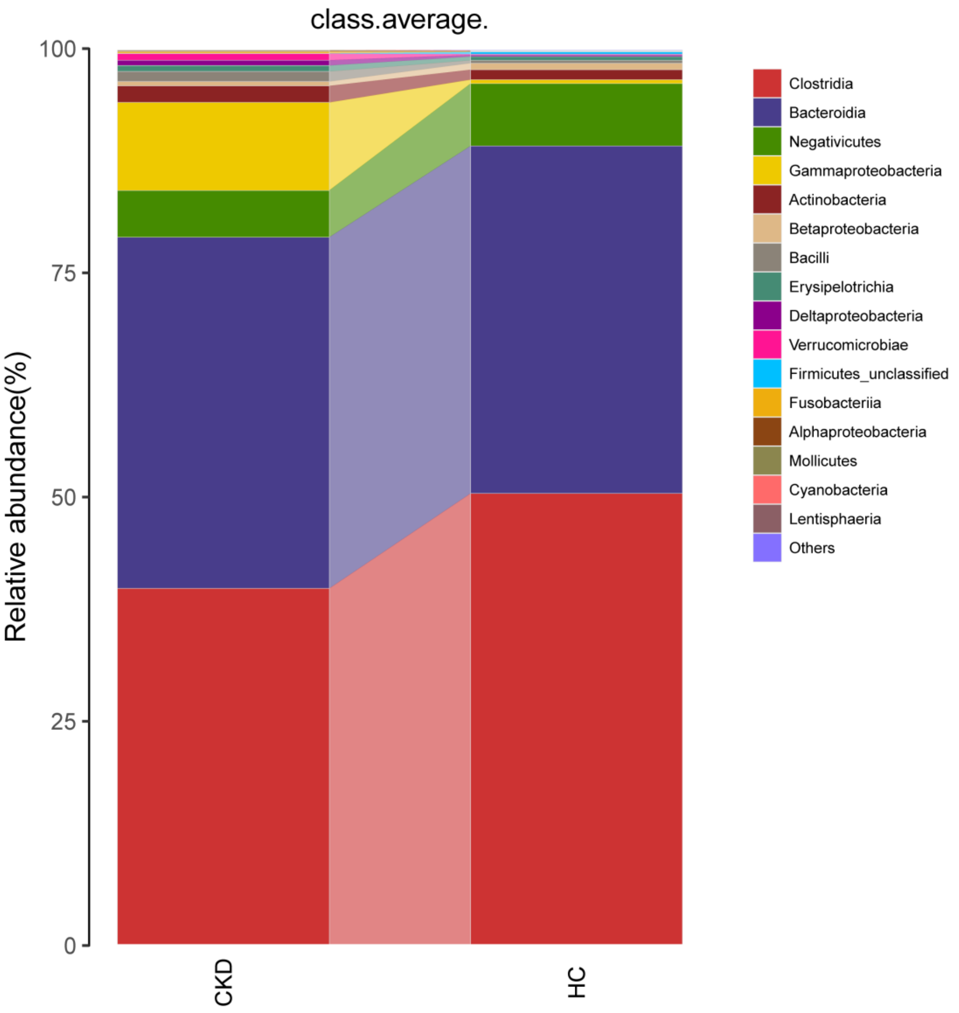

Figure S10. Average compositions and relative abundance of bacterial community in CKD (n=110) and HC (n=210) at the class level. CKD, chronic kidney disease; HC, healthy controls.

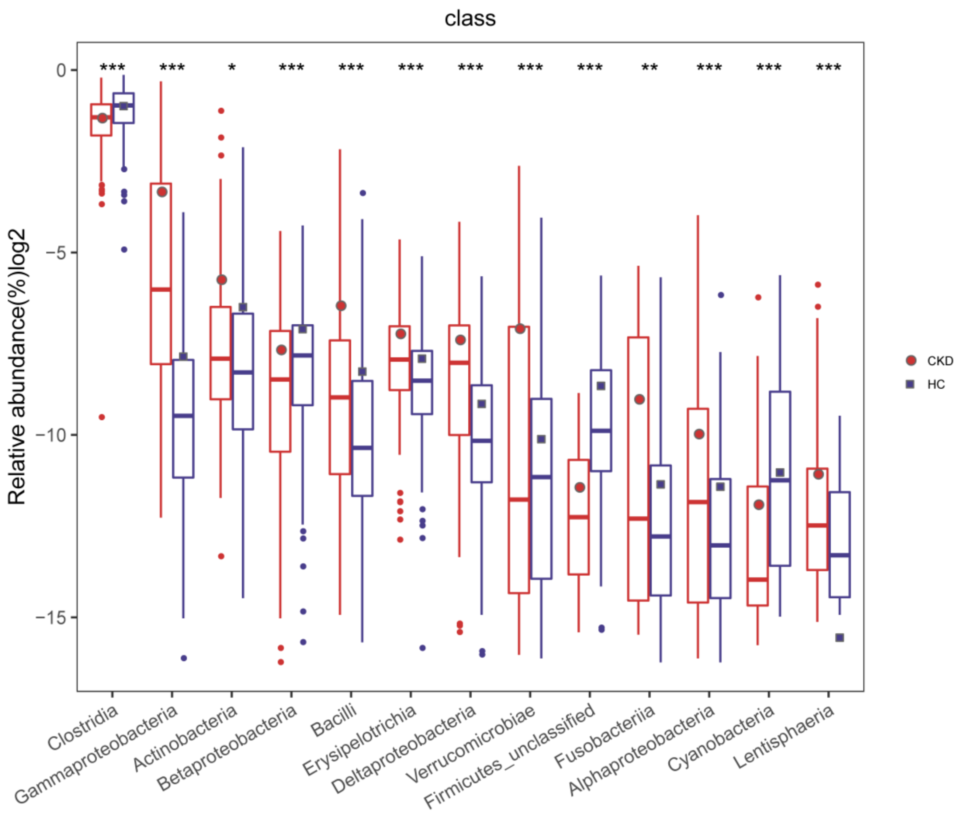

Figure S11. At the class level, 8 bacterial populations were significantly enriched, whereas 5 bacterial populations were significantly reduced in CKD (n=110) versus HC (n=210). *, *P*<0.05, **, *P*<0.01, ***, *P*<0.001. CKD, chronic kidney disease; HC, healthy controls.

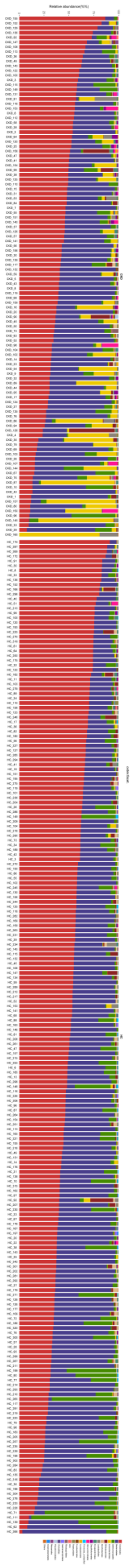

Figure S12. The composition and abundance of bacterial community at the order level in each sample of the discovery cohort (n=320). CKD, chronic kidney disease;

HC, healthy controls.

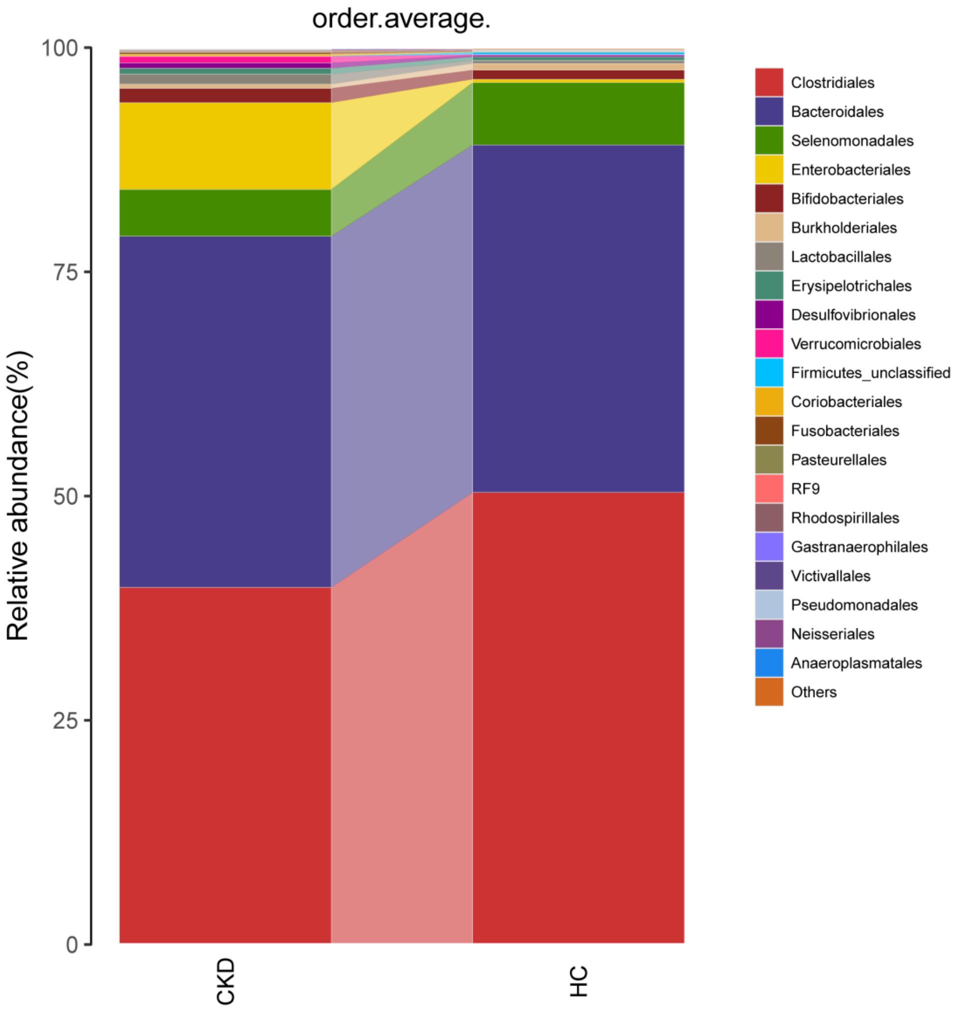

Figure S13. Average compositions and relative abundance of bacterial community in CKD (n=110) and HC (n=210) at the order level. CKD, chronic kidney disease; HC, healthy controls.

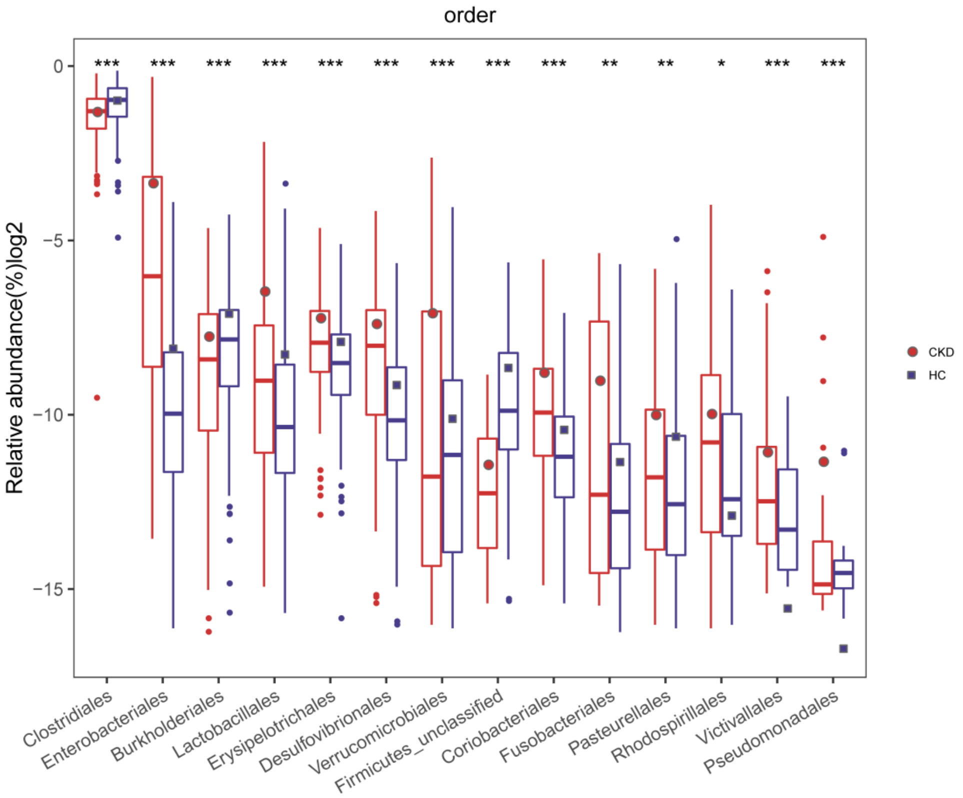

Figure S14. At the order level, 9 bacterial populations were significantly enriched, whereas 5 bacterial populations were significantly reduced in CKD (n=110) versus HC (n=210). *, P<0.05, **, P<0.01, ***, P<0.001. CKD, chronic kidney disease; HC, healthy controls.

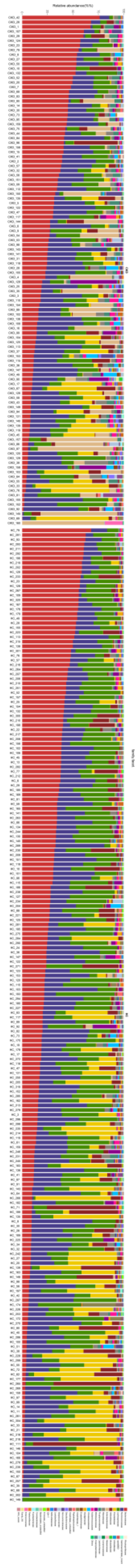

Figure S15. The composition and abundance of bacterial community at the family level in each sample of the discovery cohort (n=320). CKD, chronic kidney disease;

HC, healthy controls.

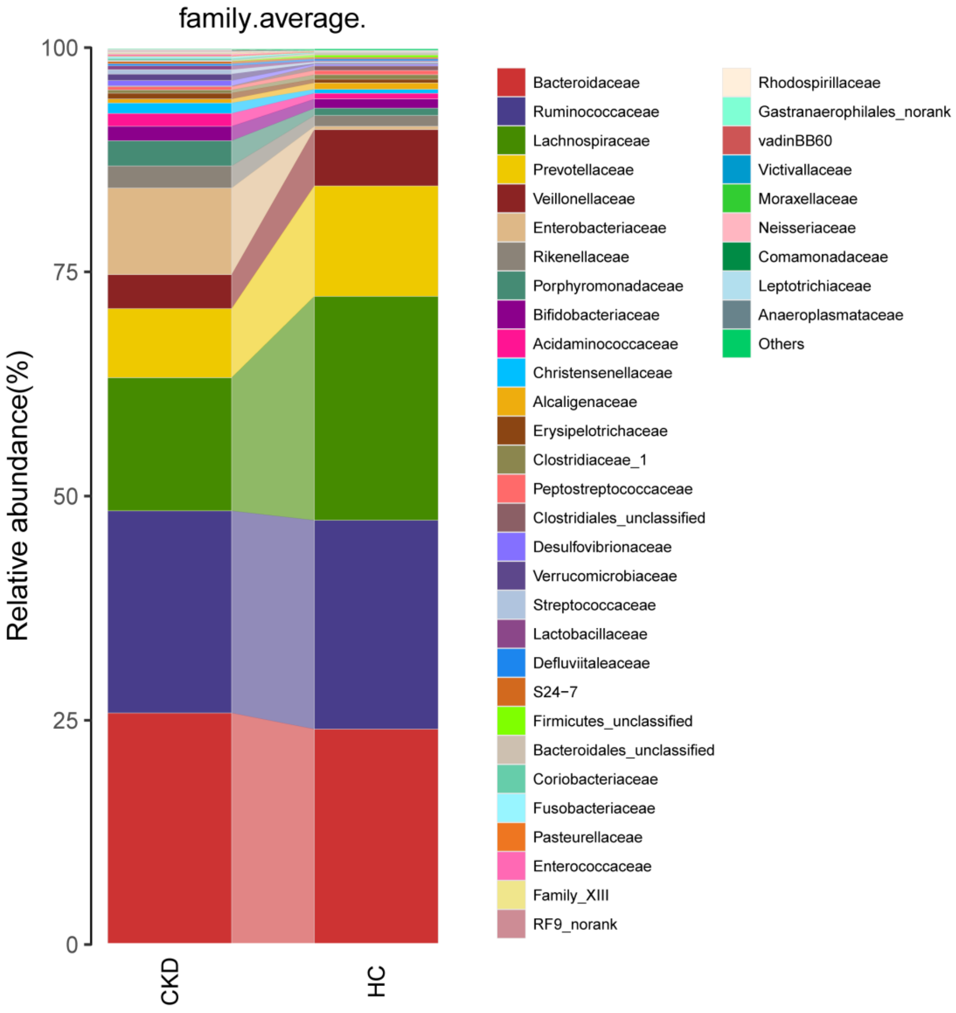

Figure S16. Average compositions and relative abundance of bacterial community in CKD (n=110) and HC (n=210) at the family level. CKD, chronic kidney disease; HC, healthy controls.

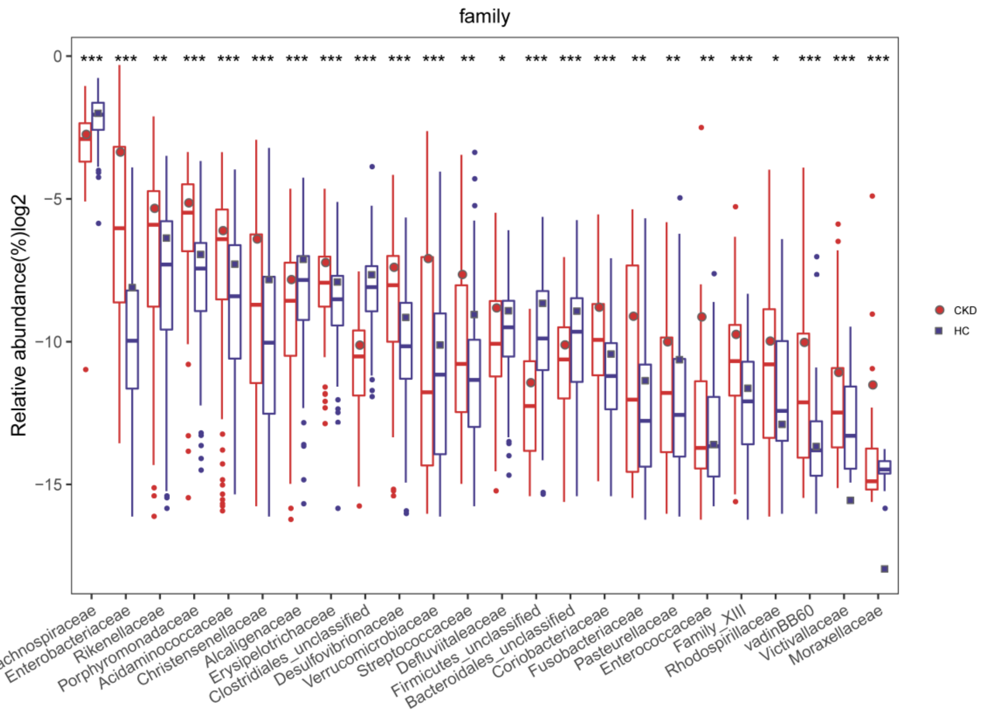

Figure S17. At the family level, 16 bacterial populations were significantly enriched, while 8 bacterial populations were significantly reduced in CKD (n=110) and HC (n=210). *, *P*<0.05, **, *P*<0.01, ***, *P*<0.001. CKD, chronic kidney disease; HC, healthy controls.

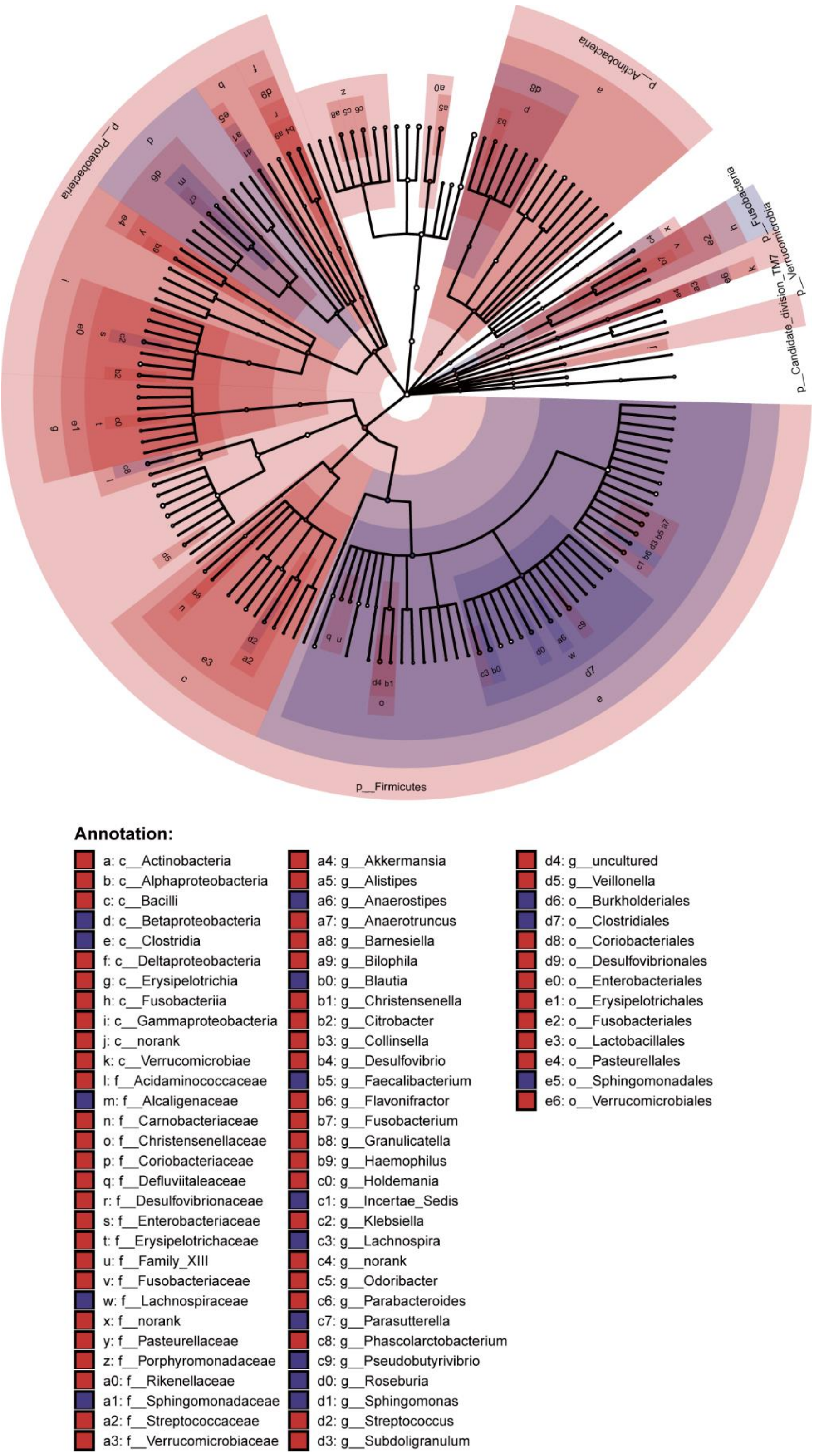

Figure S18. The phylogenetic profiles of the specific bacterial taxa and predominant bacteria associated with CKD (n=110) and HC (n=210) using the LEfSe method. CKD, chronic kidney disease; HC, healthy controls; LEfSe, linear discriminant analysis effect size.

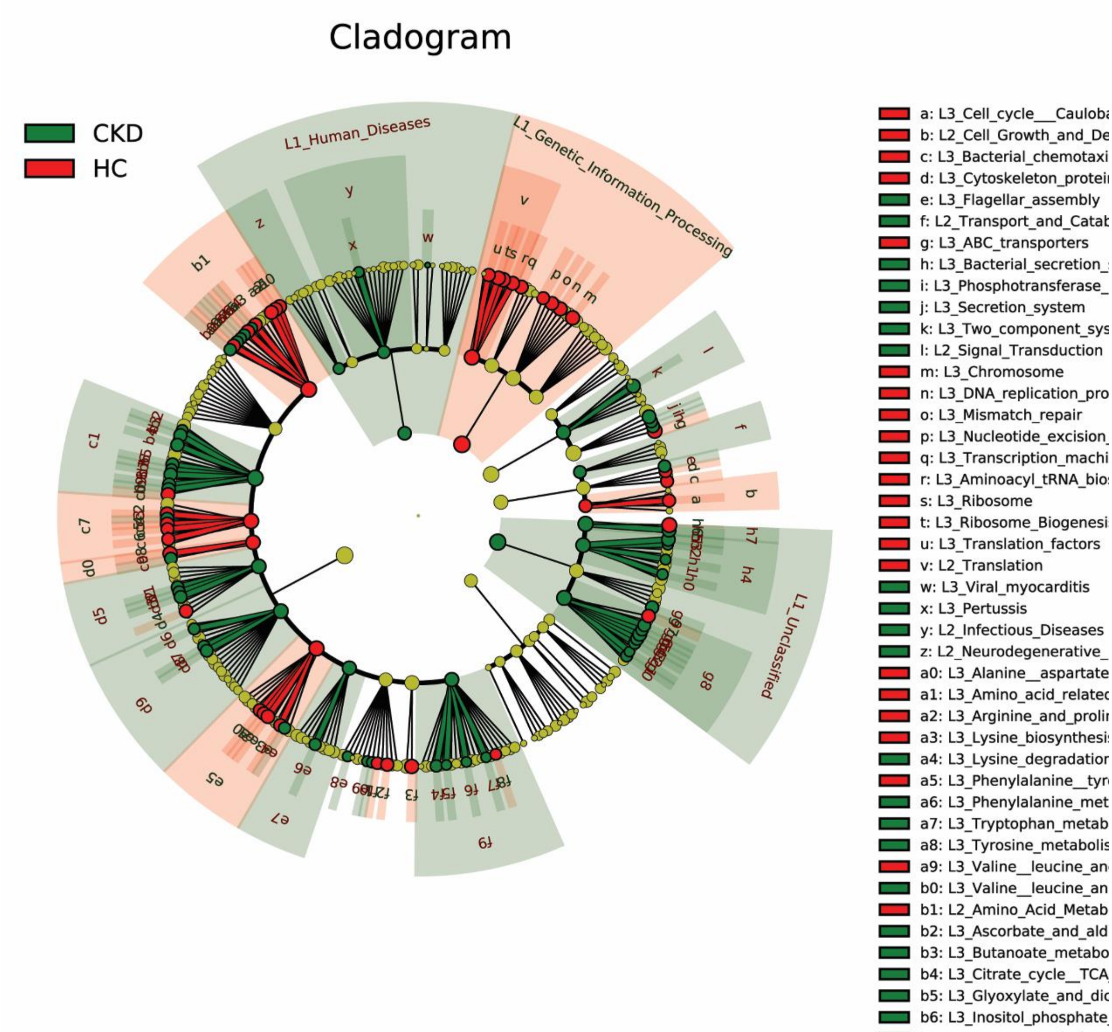

Figure S19. A cladogram showed the gut microbial community function profiles of CKD (n=110) and HC (n=210) and their predominant microbial functions. CKD, chronic kidney disease; HC, healthy controls.

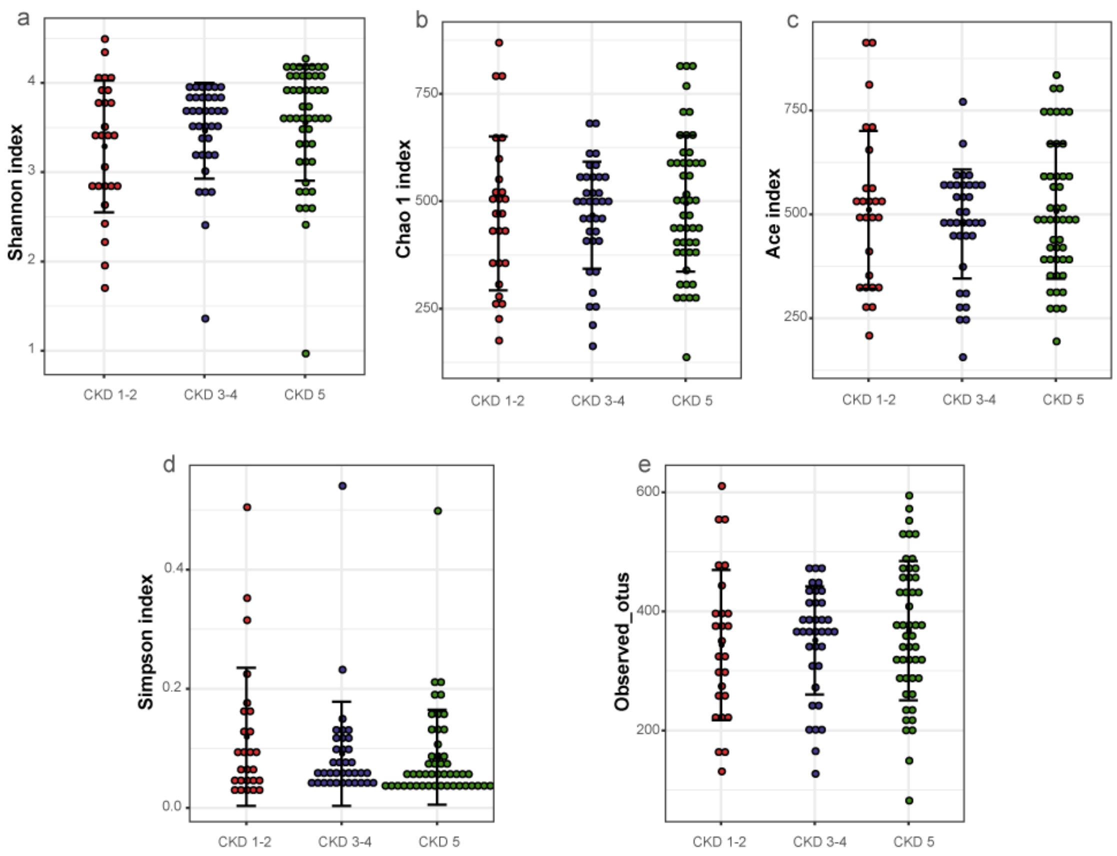

Figure S20. As estimated by the Shannon index (a), Chao 1 index (b), Ace index (c) and Simpson index (d), there was no significant difference in the gut microbial diversity of CKD stage 1-2 (n=26), CKD stage 3-4 (n=36) and CKD stage 5 (n=48) (all P>0.05). (e) The observed OTUs of CKD stage 1-2 (n=26), CKD stage 3-4 (n=36) and CKD stage 5 (n=48) was no significant difference (P>0.05). CKD, chronic kidney disease; OTUs, operational taxonomic units.

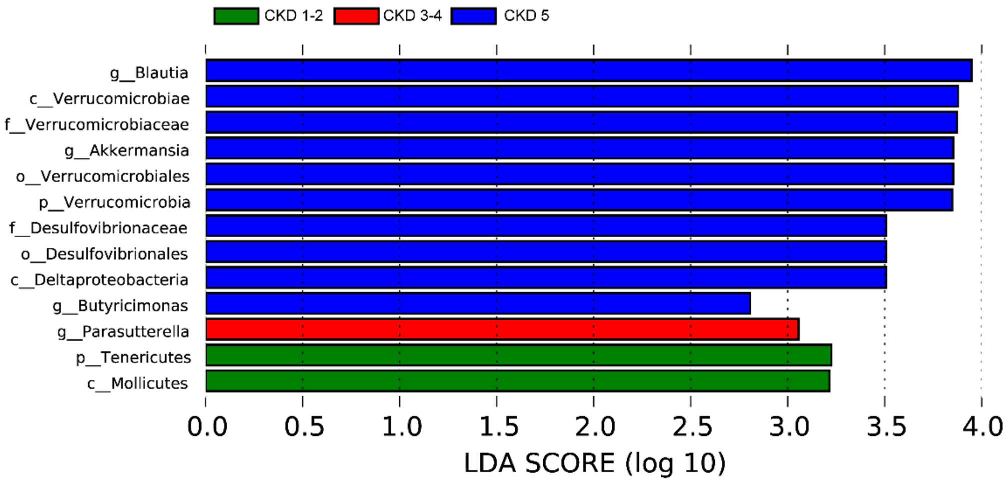

Figure S21. Based on the LDA selection, 2, 1 and 10 gut microbial taxa were enriched in CKD stage 1-2 (n=26), CKD stage 3-4 (n=36) and CKD stage 5 (n=48), respectively (all $P<0.05$). CKD, chronic kidney disease; OTUs, operational taxonomy units; LDA, linear discriminant analysis.
